# Preliminary data on the development of emotion vocabulary in typically developing children (5–13 years) using an experimental psycholinguistic measure

**DOI:** 10.3389/fpsyg.2022.982676

**Published:** 2023-01-31

**Authors:** Alexandra Sturrock, Jenny Freed

**Affiliations:** School of Health Sciences, The University of Manchester, Manchester, United Kingdom

**Keywords:** typical development, vocabulary of emotion, receptive and expressive, 5–13 years old, assessment evaluation

## Abstract

**Introduction:**

Vocabulary of emotion is integral to emotional development and emotional intelligence is associated with improved mental health outcomes. Many language disordered groups experience emotional difficulties; Developmental Language Disorders, Attention Deficit Hyperactivity Disorder and autism. However, (as in the case of autism) research tends to focus on assessing recognition of emotional states, rather than exploring labeling skills. Where labeling is assessed, measures have focused on early-acquired vocabulary (happy, sad, angry) or self/parent reporting. To date, no objective assessment has been made of vocabulary of emotion across childhood.

**Methods:**

This study uses an experimental psycholinguistic measure, The Emotion Vocabulary: Expressive and Receptive ability measure (EVER) which includes two tasks (receptive vocabulary and word generation/expressive vocabulary). This measure has capacity to demonstrate vocabulary growth across age groups. 171 participants (5.0–13.11 years) completed The EVER Measure, alongside two closely matched standardized measures of basic language: BPVS (receptive vocabulary task) and CELF (word-association task). Assessments were completed online and *en vivo* (COVID testing restrictions dependent).

**Results:**

As predicted, children’s accuracy increased on both receptive and expressive emotion vocabulary tasks, in line with age at time of testing. EVER scores were significantly predicted by age and correlated with matched basic language scores. Secondary analysis provided preliminary findings on age of acquisition for specific emotion vocabulary items.

**Discussion:**

The findings consequently demonstrate proof of concept for the use of The EVER Measure in assessing emotional vocabulary across childhood. This study provides important preliminary data on generating and recognizing emotion labels across typical child development. Critically, it extends current knowledge on emotion vocabulary acquisition into middle childhood, where linguistic ability is relatively mature. As such, findings have implications for research with potential clinical application in the assessment of older children, with either language or emotional differences or both. Findings demonstrate the need for a standardized tool, and its potential application in research and clinical practice is explored. A large-scale study offering proof of concept and reliability of The EVER Measure is indicated.

## Introduction

In recent years, there has been an accelerated interest in the relationship between linguistic and emotional development (Nuske et al., 2013; Christiansen et al., 2019; Cibralic et al., 2019; St Clair et al., 2019; Lindquist, 2021). The importance of such investigations lie in exploring associations between linguistic ability, emotional understanding and achieving better emotional outcomes (Martins et al., 2010; Roben et al., 2013; Kashdan et al., 2015). Emotional labeling has been associated with more modulated emotional responses during negative experiences (Lieberman et al., 2011), while better language skills more generally have been linked to the development of adaptive self-management strategies and fewer ‘angry’ responses in younger children (Roben et al., 2013). In addition, better labeling of emotions has been associated with improved outcomes in therapeutic interventions (Kircanski et al., 2012). Collectively the research points to a positive relationship between better skills in language/emotion language and reactive responses to emotional challenges, adoption of self-regulating strategies, and response to interventions. There are clear implications for children with known language difficulties and limitations in emotion vocabulary. Being able to identify those difficulties through dedicated assessments, could highlight specific needs of the individual and point to new pathways for intervention.

### Language and emotional understanding

Overlapping but distinct concepts of emotional understanding; including emotional competence (Saarni, 1999), emotional intelligence (Petrides et al., 2007), and emotional awareness (Lane, 2000) all entail various features related to the recognition of emotions in self and others, the ability to regulate one’s own emotions and the ability to communicate those emotions to others. As such, emotional understanding is multifactorial, and proficiency may be affected by a variety of elements, including how the individual perceives their own internal states (Craig, 2002) and attention to emotional stimuli in others (Lartseva et al., 2015). However, emotion vocabulary is generally considered integral to good emotional understanding. Saarni (1999), for example, identified the ability to use vocabulary of emotion as a core skill in acquiring emotional competence.

The exact relationship between language and emotional understanding is still under investigation, perhaps depending on theoretical viewpoints of language acquisition (Hobson et al., 2019). However, Lindquist et al. (2015) argue that a fundamental association exists between language and emotional understanding; with emotion words thought to support the acquisition of emotional concepts across childhood (Doyle and Lindquist, 2018). Indications from the wider literature suggest that language ability will predict development of emotional recognition across childhood (Pons et al., 2003) from as young as 14 months (Ruba et al., 2021). Additionally, exposure to emotional vocabulary does seem to improve emotional understanding, be it through caregiver interactions in earlier childhood (Shablack and Lindquist, 2019) or reading fiction in older children (Schwering et al., 2021). The principle that emotional knowledge is constructed through experiential learning and language development brings it in line with broader theories of abstract concept acquisition (Ponari et al., 2018). Beck et al. (2012) argue that linguistic ability is integral to developing emotional competence, perhaps due to language and particularly vocabulary, promoting emotional conceptualization. A tool for objectively exploring emotion vocabulary could shed further light on this relationship. Research also indicates a gradual refining of emotion language and concepts across childhood (Grosse et al., 2021) and into adulthood (Nook et al., 2020). This includes better definitions of emotional categories and developing a broader range of emotional labels with which to describe emotions (Bazhydai et al., 2019; Grosse et al., 2021). It is likely that this continuing refinement of emotion concepts is important when establishing more complex and ambiguous emotional responses pertinent to adult emotional contexts and social interactions. Therefore, it is essential that any tool aiming to evaluate emotional understanding, in relation to emotional labeling, has the capacity to do this across childhood and into adolescence.

### Implications for clinical populations

Given the relationship between emotional understanding and language ability, it would be expected that individuals with language disorders might be particularly at risk of poor outcomes of emotional well-being. In fact, children with Developmental Language Disorders (previously referred to as specific language impairment) are known to have elevated rates of emotional and behavioral difficulties (Goh et al., 2021) and higher rates of mental health problems across the life-span (Schoon et al., 2010). Autistic individuals are also more likely to experience mental health problems, such as anxiety and depression (Hofvander et al., 2009) and difficulties with emotional understanding are well-documented for this group (Bird and Cook, 2013). Language abilities in autism are heterogeneous ranging from non-verbal to highly advanced (Kjelgaard and Tager-Flusberg, 2001). However, several studies indicate differences in autistic individuals’ understanding and use of emotional language, despite preserved higher-linguistic ability. For example, labeling internal states in others (Kauschke et al., 2016), retelling emotional memories (Goddard et al., 2014), receptive word knowledge (Sturrock et al., 2019), and use of emotion referents in spontaneous dialog (Adams et al., 2002), might all be limited in this group. Other factors are also associated with better emotional understanding in clinical populations, such as poorer perspective taking (Mazefsky and White, 2014) or reduced facial recognition (Merkenschlager et al., 2012). In recent years a growing body of work has focused on alexithymia, a cluster of cognitive and behavioral traits, characterized by difficulties identifying and describing ones emotions (Hobson et al., 2019). Better scores on alexithymia measures have been associated with improved emotional understanding in groups with autism (Bird and Cook, 2013), Developmental Language Disorders (Hobson and van den Bedem, 2021) and Attention Deficit and Hyperactivity Disorder (Edel et al., 2010). This might indicate that alexithymia mediates emotional understanding in these groups. However, there are flaws with existing measures of alexithymia, which are typically based on self-or parent report and tend to focus on different features of this condition (Hobson and van den Bedem, 2021). Objective measures of emotion vocabulary could help unpick the complex interactions of emotional vocabulary and understanding both within this population and elsewhere in clinical populations.

Although language difficulties in clinical groups are associated with poorer outcomes on emotional wellbeing measures, the direction of causation is not clear (Forrest et al., 2021). Emotional dysregulation is common in the language disordered groups previously mentioned (Fujiki et al., 2002; Graziano and Garcia, 2016; Conner et al., 2021), which may in itself influence the development of emotional concepts. In addition, language and communication difficulties are associated with reduced quality of friendships (Fujiki et al., 2001), educational (Snowling et al., 2001) and employment outcomes (Clegg et al., 2005), which themselves will have an impact on emotional wellbeing. A measure of emotion vocabulary could provide new information about a key aspect of language profiles in clinical populations and allow the exploration of the relationship between emotional vocabulary development and emotional regulation. Understanding typical development of emotion vocabulary in middle childhood, will also provide data for comparison with older children who experience language difficulties.

### Current measures of emotion vocabulary

To date, researchers have sought to measure emotion vocabulary in a number of ways. Several studies have asked participants to explain the definition of emotion words, with their responses rated against a set of criteria (Greenberg et al., 1995; Kelly et al., 2004; Bauminger et al., 2005). The Kusché Affective Inventory (KAI: Greenberg et al., 1995) has been used in a number of these studies, and focuses on a small subset of relatively mature emotion concepts: proud, guilty, jealous, nervous, and lonely. Nook et al. (2020) expanded on this method to explore emotion word knowledge in a wider range of ages across childhood. They established a repertoire of 27 emotion terms, reflecting dimensions of positive, neutral and negative valence and varying levels of arousal. Items ranged from early basic emotions through to those arising later in development. The individual was asked ‘what does xx mean?’ with results indicating that definitions of emotion vocabulary were finessed throughout childhood and into adulthood. This type of methodology allows a detailed analysis of an individual’s higher level emotion vocabulary. However, due to the responses being descriptive in nature, this form of assessment relies heavily on good levels of general language ability.

The KAI measure also includes a subtest for the generation of emotion words. Beck et al. (2012) used this to compare emotion vocabulary to general language competence in a cohort of mixed-age children, demonstrating a strong positive correlation between the two. A variation of this used by Bazhydai et al. (2019) asked adolescent participants to generate words within a basic emotion category, for example, given the early emerging category of ‘happy’ could they then generate other later emerging synonyms for that word; e.g., joyful, ecstatic, exuberant. This was repeated for four other emotional states; relaxed, angry, sad and nervous. This method of data collection was used to demonstrate a breadth of emotion vocabulary developing across adolescence.

Several studies employed story vignettes to elicit spontaneous labeling of characters’ emotion. In Grosse et al. (2021), participants were introduced to vignettes depicting a character experiencing joy, fear, sadness, anger, disgust and surprise and were asked to describe the characters’ emotional state with a single word. In an adaptation of this, Beck et al. (2012) included vignettes exploring mixed emotions. In combination, these assessments have been used to explore emotional understanding and emotional labeling skills in a range of child ages. Fewer studies have attempted to measure expression of emotion in response to real emotional events (Lieberman et al., 2011; Kircanski et al., 2012). While this offers an important window into self-reporting skills there are clearly ethical implications for exploring the reporting of negative emotions.

Comprehension of emotion vocabulary has also been investigated in typically-developing children through completion of a 336 emotion word checklist (Baron-Cohen et al., 2010). In this study, parents were asked if their child (aged 4–12) understood words on the checklist, while for children aged 12–16 the checklist was completed either by the parent or child. This study provided data (*n* = 377) across a range of ages, and demonstrated a trajectory of emotion language development across childhood. However, there are limitations when interpreting data collected through self- and other- report. Not only are biases likely but also deficits in a person’s comprehension of language are notoriously hard to detect by a third party (Conti-Ramsden and Durkin, 2012). A more robust measurement would include direct assessment of this knowledge.

While assessment of emotion vocabulary has more recently included stimuli to account for a range of abilities, there is still a tendency to focus on basic vocabulary or at least a small range of vocabulary items. Where higher level vocabulary of emotion has been investigated it has typically relied on report measures or tasks which require a high degree of general language skills, potentially confounding the results. Therefore, an important step in understanding the childhood development of emotion vocabulary, would be to explore expressive and receptive skills on a range of emotion vocabulary items (identified in the literature). This would most effectively be conducted through direct assessment in a large sample of children representative of various age groups across childhood. Findings will contribute to understanding of the development of emotion vocabulary across childhood and provide some age-based normative data for comparisons to clinical groups. It is hoped that The EVER (Emotion Vocabulary: Expressive and Receptive) Measure can be used as a direct assessment to help unpick the complex relationship between language of emotion and emotional language development in clinical groups, isolating specific areas of need and leading to the development of dedicated interventions.

### Current study aims

The current research aims to: (1) evaluate the use of The EVER Measure as a tool capable of assessing the development of emotion vocabulary across childhood; (2) provide preliminary data on the age of acquisition for a subset of emotion vocabulary represented in The EVER receptive measure and spontaneously generated emotion vocabulary using The EVER word association task; (3) scrutinize data for evidence of factors that will inform future development of The EVER Measure. Therefore, research questions can be outlined as such:

1. Does The EVER Measure demonstrate proof of concept as a tool capable of assessing the development of emotion vocabulary across childhood?
2. What preliminary data can The EVER Measure (receptive and WA task) contribute to the wider understanding of emotion vocabulary acquisition across childhood (5–13.11 years)?
3. How do findings from this current study inform the future development of The EVER Measure?

## Materials and methods

The EVER Measure is comprised of two parts, the Receptive Emotion Vocabulary (REV) task and the Word Association – Emotion category (WA-Emo) task. The original language of emotion measures (henceforth called The EVER Measure) were developed to test the language and communication abilities of autistic children without learning disability compared to non-autistic peers (see Sturrock et al., 2019). Full details are provided below.

Data for the current study were collected from three distinctive cohorts, (1) control participants within a group comparison study (Sturrock et al., 2019); (2) a purposive sample group identified for stage one piloting of The EVER Measure; (3) participants purposively recruited to represent a *minimum* of *n* = 16 children per year group (aged 5–13:11) when combined with cohorts (1) and (2). Details are provided below. Cohorts 1 and 2 were seen in a face to face testing environment and completed a broader range of measures than is included here, while cohort (3) were assessed on a dedicated set of test items pertinent to this study in an online setting (conferencing software platform Zoom). Changes to testing procedures were due to constraints arising from the Covid pandemic (2020–22). Each stage of data collection obtained its own ethical approval. Details of materials used, ethical approval and protocol are discussed below.

### Participants

All participants (*n* = 171) met the inclusion criteria; English as a first language; attendance at a mainstream school in the United Kingdom without educational support; no diagnosis of language or communication difficulties which would affect communication (including those of physical, cognitive or sensory-neural etiology). These were determined through a combination of parental report and researcher assessment during testing sessions, e.g., using the British Picture Vocabulary Scale (BPVS; ed. 2 Dunn et al., 2003; ed. 3 Dunn et al., 2009): cut off = standard score ≥ 70. The overall group of 171 participants comprises the following subsets of participants:

*Cohort 1:* 24 participants (*n* = 11 girls and n = 13 boys) aged 8:11 to 11:06 years. Purposively recruited to act as typically developing controls against a clinical autistic group matched on age and sex. They were recruited from the North West United Kingdom, observationally determined to represent a range of social economic status (SES) groups (but with a predominance of middle-high). Specific data were not collected on this point. Parents contacted the research team after receiving the advert and parent information sheet from The UK ESRC International Center for Language and Communication Development (LuCiD) research group and database. Consent and assent was taken prior to testing. Participation was face-to-face at home or in school.

*Cohort 2:* 48 participants (*n* = 34 girls and *n* = 14 boys) aged 7:04–10:04 years. Purposively recruited from school year groups three, five and seven, directly through two state primary and one secondary school in North West United Kingdom. As an indicator of social economic status, the schools experienced below the 14.3% average rate of free school meal (1.4–10.6%) according to https://www.get-information-schools.service.gov.uk/ accessed 2016. Advert and information sheets were distributed by teachers and consent and assent was taken and returned to researchers through the school. Participation was face-to-face in school.

*Cohort 3:* 99 participants (*n* = 41 girls and *n* = 58 boys) aged 5:0–13:09 years. Purposively recruited to meet participant thresholds of no fewer than eight girls and eight boys per age bracket (5:0–5:11 through to 13:0–13:11 years) when combined with child data from cohorts (1) and (2). Demographic data on SES was not collected. Despite participants being recruited widely across the UK, observationally there was a skew to middle and high SES and North West Locality. Parents contacted the research team after receiving the advert and parent information sheet from The ESRC International Center for Language and Communication Development (LuCiD) research group and database or *via* social media advertisement (e.g., on mum’s net, etc.). Consent and assent were sent electronically and returned directly to researchers *via* email. Participation was online.

### Procedure

*Cohort 1*: Children were seen individually at their home or school. One parent was asked to attend the first session to give informed consent and to verify their child met the inclusion criteria. Two subsequent sessions lasting ~60 min focused on undertaking a battery of 15 direct assessments including those reported here. For full details of the original test procedure please see Sturrock et al. (2019). All test items were undertaken in a fixed order. Critical to this study this included: (1) BPVS-2, (2) REV, (3) then test break ~20 min including administration of an unrelated language assessment not reported here, (4) Word Association (WA) task, (5) WA-Emo. These activities were all undertaken within session two of testing. Ethical Approval was gained from South West – Central Bristol NHS Research Ethics Committee (November 2015).

*Cohort 2*: Participants were seen individually in school during lesson time, in an area shielded from distractions. One session lasting ~30 min included the following assessments in this order (1) WA task, (2) WA-Emo, (3) BPVS-2, (4) REV. Ethical approval was received from The University of Manchester Ethics Committee and The University of Bath Department of Psychology Ethics Committee.

*Cohort 3*: Children were seen within a Zoom conferencing platform. Parents were asked to ensure the child was in an area free from disturbance for the duration of the session. Parents were asked to be available during this time in case of technical difficulties, but were advised to let the child answer all questions for themselves without offering support. All parents complied with these requests. The session had differentiated timings depending on the age of the child. Children aged 5 and 6 years old would automatically be assessed in two 30-min sessions. Children aged 7 years and above would typically be assessed in one 40-min session, with the option for a split session (as described for 5 and 6 year olds) on request. Breaks were offered to all and were taken up by many of the younger children. Any child could request to stop a session and re-start on another day, although there were no occasions where this occurred. The order of test materials was (1) BPVS-3, (2) WA task, (3) WA-Emo, (4) REV. Ethical approval was received from The University of Manchester Ethics Committee.

*Justification of procedure changes*: The REV task moved from being the second test item in procedure 1, to being the final test item in procedures 2 and 3. In protocol one, REV and WA-Emo was separated by at least a 20 min gap in emotion language testing which included a complex sentence level language task. This could not be replicated in the shorter session so the order was changed to avoid priming the child with emotion language prior to testing word recall on this category. The BPVS was updated from edition 2 to edition 3, in line with publisher recommendations. In addition, it moved position between procedures. Although there are no confounding factors determining where this test item comes, in the final iteration it moved to initial position for administrative reasons. As a longer test item (~15 min) it was felt that it could be administered in isolation, should the session need to be split in two. This would leave the three shorter items in the follow up session and avoid splitting consecutive administration of The EVER Measure. This means we had three testing cohorts of children who had slightly different routes to recruitment, demographic distribution and testing procedures.

The procedure for established tasks was derived directly from published guidelines. The procedure for experimental measures are detailed below.

### Materials

#### Basic language measures

- *British Picture Vocabulary Scale* second edition (BPVS-2; Dunn et al., 2003) and *third edition* (BPVS-3; Dunn et al., 2009): the child demonstrates receptive word knowledge by identifying a target word from a choice of four pictures following a spoken presentation of the word target by the assessor. There are a maximum of 168 test items of increasing complexity. In the on-line testing procedure the visual stimuli was presented *via* screen share, using slides of the pictures from the BPVS booklet. No other details of administration were adapted for online testing. Raw scores were generated and are presented in the results section along with standard scores.
- *The Clinical Evaluation of Language Fundamentals – fourth edition* (CELF-4, UK version): *Word Associations* (WA) *subtest* (Semel et al., 2003): The child is asked to generate words within super-ordinate categories of animals, food, and occupations, following the instruction: “Name different jobs or occupations that people might have. Name as many as you can in 1 minute. For example, you could say babysitter or mechanic. Now you name some more. Start now.” Audio recording was taken of the child’s responses in line with publication guidelines. During the online testing session this was of greater importance to avoid items potentially lost through internet speed dips. However, no data was compromised in this way.
  Raw scores were generated for each category, one point for each correct item. Scoring was adapted from publisher recommendations to increase inter-rater consistency and a scoring matrix was devised (see below for details). Development of this is discussed below.

#### The EVER Measure

**Figure 1:**
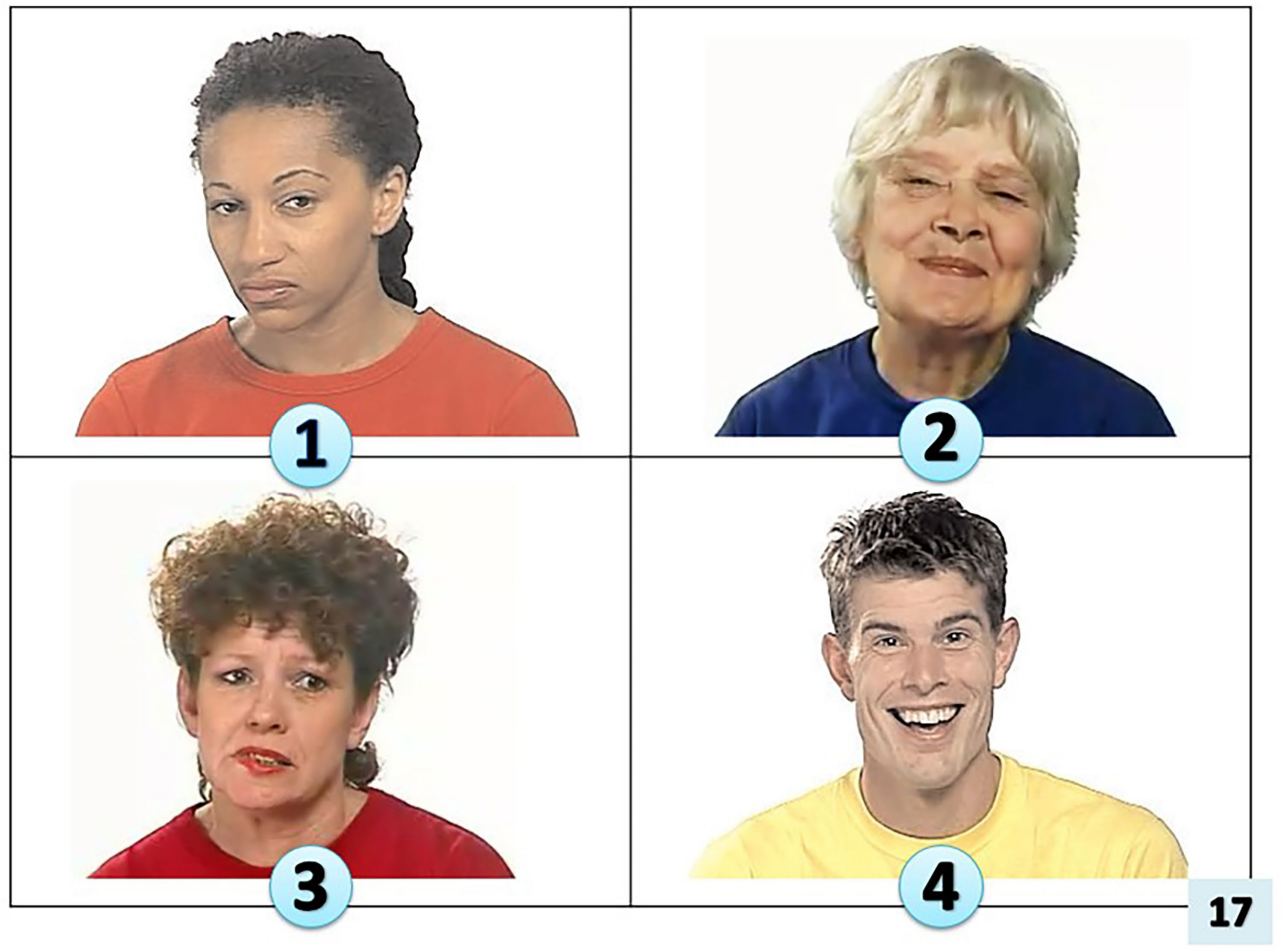
Picture stimuli for Receptive Emotion Vocabulary (REV) task (images, with the permission of The Autism Research Centre, The University of Cambridge; see footnote 1).

- *Receptive Emotion Vocabulary* (REV): a novel measure (using images with the permission of The Autism Research Centre, The University of Cambridge)^1^ by which the child identifies a target emotion vocabulary item from a choice of four pictures (see Figure 1), following a spoken presentation of the word target by the assessor. Thirty-nine test items were developed to cover the receptive vocabulary of emotion over a range of ages and language abilities. Responses were scored one point for each test item correct and it was noted whether incorrect choices fell into the close or distant distracter categories. The latter two items demonstrated broad understanding versus lack of understanding of the target word. Full details of test development are provided below.
- *Word Association – Emotions Category* (WA:Emo) *task:* an experimental task which broadly follows the protocol used in Beck et al. (2012) and CELF-4 (Semel et al., 2003). Using emotion as the super-ordinate category, it was developed to measure the parameter of expressive emotion vocabulary and word association. The instruction is to “Name different feelings or emotions that people might have. Name as many as you can in 1 minute. For example, you could say *happy* or *embarrassed*. Now you name some more. Start now.” Audio recording was taken of the child’s responses as standard. During the online testing session this was of greater importance to avoid items potentially lost through internet speed dips. However, no data was compromised in this way.
  The task generates raw scores, one point for each correct item. However, to increase inter-rater consistency a scoring matrix and emotion database was devised to support the scorer to make decisions about correctness. Development of this was critical to advancing consistency within The EVER Measure scoring protocols, and is discussed below.

### The EVER Measure development

*REV stimuli:* the Receptive Emotion Vocabulary task was designed to emulate other receptive language measures (for example, the BPVS), with the administrator presenting a target word and the participant selecting the correct answer from a choice of picture stimuli. In this way expressive language skills are minimized/eliminated within the task. In this instance, the authors also wanted to identify other variations in response. To achieve this each page of the picture stimuli had one picture depicting the ‘target word’ one depicting a ‘close distractor’, and two ‘distant distractors’ (detailed below).

In the first stage of development, the list of target words (*n* = 37) was established with reference to a database of emotion vocabulary items (*n* = 336) which included details of reported age of acquisition for a large number (*n* = 377) of participants (Baron-Cohen et al., 2010). In the original data, items were operationalized as ‘established’ for a specific age group if they were reported as acquired by ≥ 75% of participants. The full list was then filtered to remove items which required an action (e.g., giving), the involvement of another individual (e.g., protective) or the opinion of another person (e.g., liked). This resulted in 215 potential word items. The remaining list was filtered to remove items which could have a literal alternative translation (e.g., bitter or cold), in order to remove confounding factors when testing individuals with difficulties interpreting non-literal meaning. The remaining words were tabulated reflecting their reported age of acquisition and grouped by synonym (Baron-Cohen et al., 2010). For instance ‘happy’ was established at 4–6 years of age, its synonym, ‘cheerful’, was understood at 7–8 years, and ‘bliss’ at 11–12 years. Using this table a further set of words were eliminated by removing any synonyms thought to emerge within the same age bracket (of 2 years). Synonyms were not permitted in consecutive age brackets to remove priming effects.

In the end, 37 target words were generated, with ~5 representing each age bracket (5–16 years). Ekman’s six basic emotions (1992) were included (happy, sad, afraid, angry, surprised, disgusted). Synonyms of this basic set were then represented with an item at a later age of acquisition. Each age bracket included a representative distribution of valence (positive, neutral, negative) with a skew toward more negative words, which reflected the original data set. Target words were matched to images from the Autism Research Centre (The University of Cambridge; see footnote 1). Each item of REV stimuli depicted four images, target, close distractor and two distant distractors. Close distractors were chosen from items of the same age of acquisition and valence, but not synonym category. Of the two distant distractors, one was from the same age of acquisition but not from the same valence, the other was randomly allocated but not meeting the definition of a ‘close’ distractor. Close distractors were noted in the scoring sheet. A full set of target and close distractors can be found in appendix 1.

Stimuli were organized in chronological order according to the age at which the target word was expected to emerge (according to Baron-Cohen et al., 2010). The target and distractor images themselves were randomly allocated on the page. The resulting stimuli was trialed with naive adults (*n* = 10) with open questioning following testing to establish any difficulties with either the target images or distractors. Minor alterations were made following this process, for example, if the target and close distractor were too similar.

**Figure 2:**
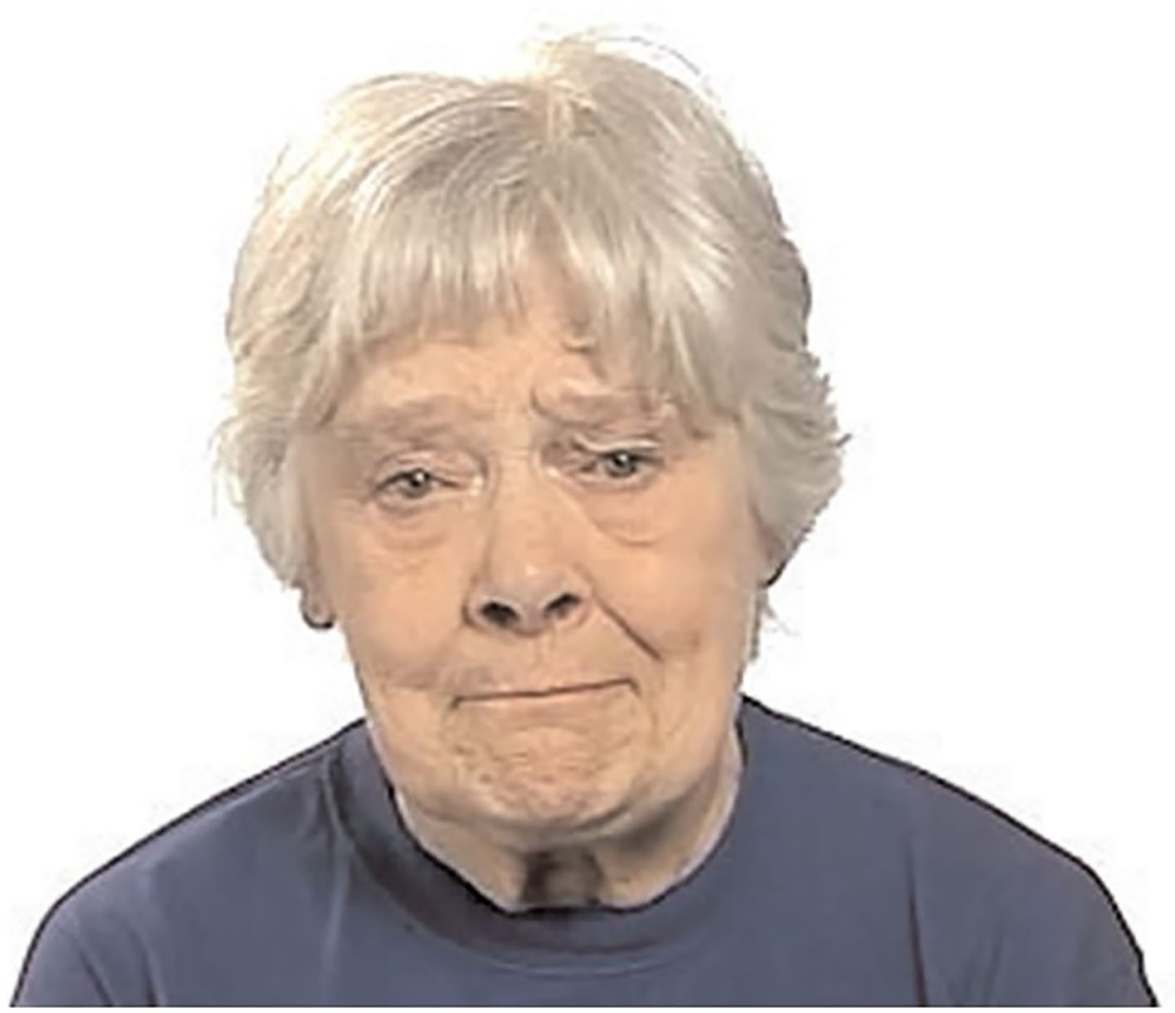
Picture stimuli for target word *worried_2.* Reproduced with permission from The Autism Research Centre, The University of Cambridge; see footnote 1.

**Figure 3:**
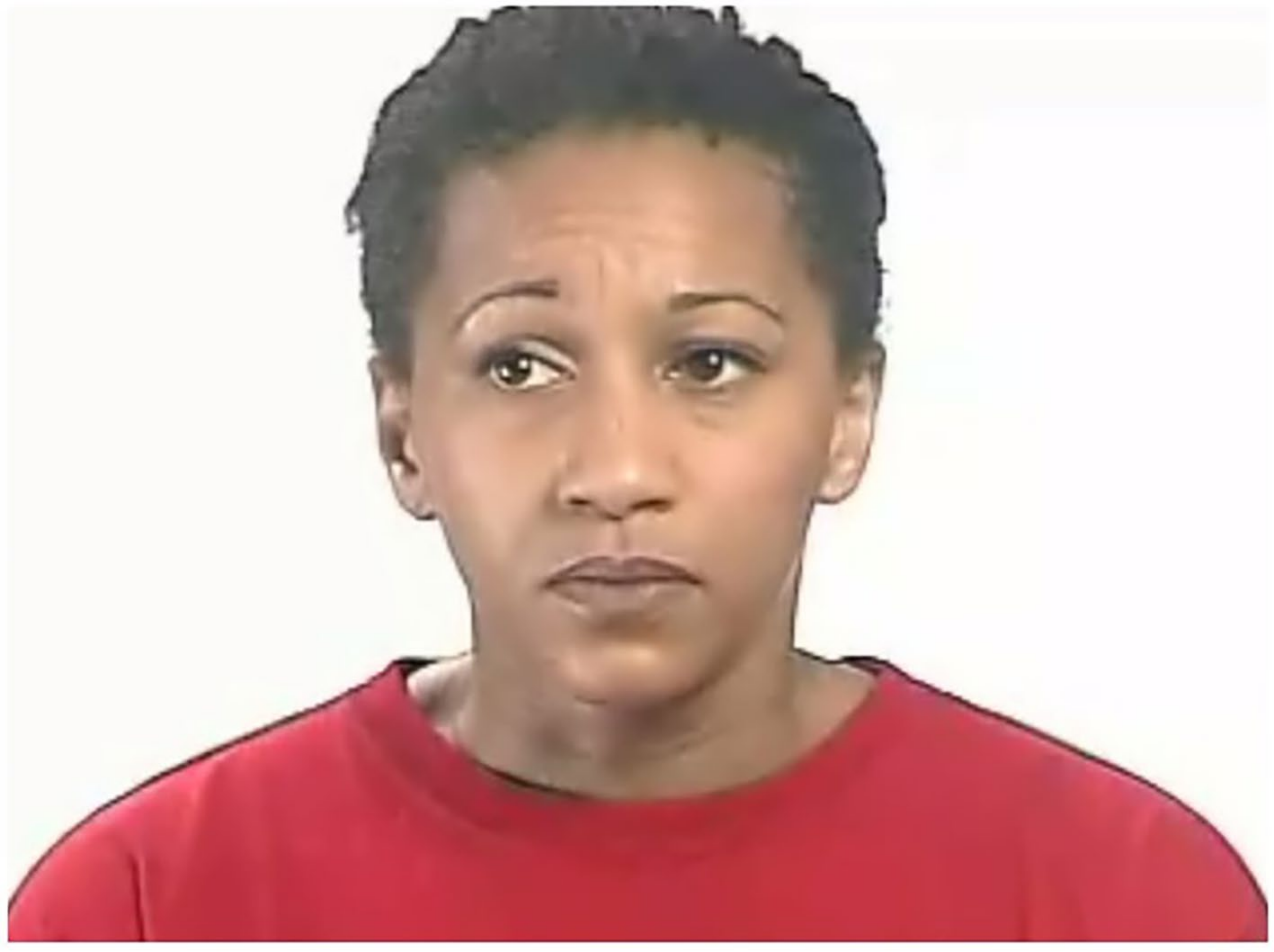
Picture stimuli for target word *worried_1.* Reproduced with permission from The Autism Research Centre, The University of Cambridge; see footnote 1.

Following this pilot, two duplicated words were included to test particular hypotheses about stimuli. The word ‘worried’ was included as two test items. In the first instance, ‘worried_1’ (see Figure 2), the picture stimuli for the target word was judged by pilot study participants to be easily discriminated from the close distractor. In the second instance, ‘worried_2’ (see Figure 3), pilot participants had judged that the picture stimuli was less easily discriminated from the close distractor.

Similarly, picture stimuli for ‘threatened_2’ were included in one instance alongside non-confounding distractors (close and distant). In the other instance, ‘threatened_1’, the stimuli was placed alongside a picture that represented the emotion ‘threatening’. This is potentially confounding, as children with a morphological language or even speech perception difficulty may be less confident choosing the correct picture stimuli. Although this study was interested in typical language of emotion development, these two items were included to explore any confounding effects on responses. Duplicate test items were positioned in non-consecutive order.

In testing cohorts 1 and 2 all children completed all test items on the REV. But for testing of cohort 3 a discontinuation rule was imposed to take into account testing with younger children. The discontinuation rule was to be applied after five consecutive incorrect answers (this included responses of ‘do not know’). For parity, this rule was retrospectively applied to data collected from the previous data sets.

*WA:Emo scoring matrix:* WA data for cohorts 1 and 2 were originally scored using rules of administration from CELF-WA and derivations of these rules were applied to the WA:Emo task. However, in this current study a higher degree of exactitude was desired. To this end, a matrix was established for each of the word categories: animals, food, occupations (appendix 2) and emotions (appendix 3). This was applied to cohort 3 data and retrospectively applied to cohort 1 and 2 for parity. The process for defining scores for the emotion category also resulted in a database of word items. The emotion matrix development will now be discussed as an exemplar.

As a first step, a definition of ‘emotion’ was generated with reference to the literature. This definition was applied to five sets of participant responses by three independent researchers, assigning each word item as correct or incorrect and generating a total number of correct items. Where independent scorers generated different results the word was discussed to establish agreement. Simultaneously, a set of scoring rules were generated through this discussion, for example,

‘Emotions can vary in typicality; ‘fear’ is more typical than ‘awe’, but both are correct for the purposes of scoring this project’

Through an iterative process these rules were then applied to several subsequent rounds of independent scoring, with each round resulting in further discussion and increased specificity of the matrix. As each round of discussion occurred a catalog of words was also generated. This represented items that had been deemed as correct after having undergone consideration by the group and applying the matrix. For full and final scoring criteria see appendix 3.

At this point assessment for inter-rater reliability was undertaken between the two primary scorers. Cohen’s ᴋappa was conducted to determine the level of agreement between two raters scoring correct or incorrect on a novel set of emotion category words (174 items from 21 participants). A strong agreement was found (*ᴋ* = 0.729 (95% CI, 0.570 to 0.888), *p* < 0.0005).

The updated marking criteria was then retrospectively applied to data for all three cohorts, before analysis was undertaken.

A similar process was undertaken for the other WA categories (see appendix 2), including establishing inter-rater reliability, which ranged from moderate to strong [food: *ᴋ* = 0.435 (95% CI, 0.172–0.698), *p* < 0.0005], [occupations: *ᴋ* = 0.775 (95% CI, 0.634–0.916), *p* < 0.0005, animals: *ᴋ* = 0.976 (95% CI, 0.929–1.023), *p* < 0.0005].

### Analysis

Psychometric analysis directly addresses the research questions. The primary aim is to provide proof of concept for The EVER Measure, and the analysis methods chosen allow a detailed review of measures on an item-by-item basis in preparation for future development.

1. Proof of concept will be ascertained by (a) using linear regression to demonstrate a relationship between total correct scores on The EVER Measure and age, (b) using correlations to explore pattern of acquisition on emotion language vocabulary as established by The EVER Measure with matched standardized assessments of general language.
2. Preliminary data on emotion vocabulary acquisition across childhood (5–13.11 years) will be analyzed using counts and percentages of word items occurring correctly in both the receptive language and the word association task. This will be compared across age groups to ascertain patterns of emergence.
3. Informing future development of The EVER Measure, will be undertaken by offering a close scrutiny of findings. This will include comparisons of data sets and exploring anomalous findings therein.

We anticipate that The EVER Measure will have the capacity to identify growth in the emotion vocabularies of typically developing children between 5 and 13 years. We expect that preliminary data will demonstrate emotion vocabulary emergence, both in receptive and expressive skills, in line with age and basic vocabulary. We expect to find anomalous data that can inform evidence-based revisions of the measure before undertaking future research.

## Results

171 participants were recruited (*N* = 86 females, *N* = 85 males). Table 1 shows descriptive details concerning the distribution of females and males, participant numbers, cohort (see methods for implications), testing procedure (online or face-to-face) and number of testing sessions according to age groupings. There was no statistical difference between number of female and male participants within any age group (*t* (169) = 0.93, *p* = 0.829). However, there were known differences in terms of testing procedures, e.g., protocols, number of test sessions (younger versus older children) all of which are outlined in the methods section. Number of testing sessions were not hypothesized to affect scores. However, testing cohorts (1–3) were included as independent variables in the regression analysis (detailed below) to identify any influence on scores.

**Table 1:** Showing descriptive details, by age group, of participant numbers, sex, cohort inclusion, testing procedures and number of testing sessions.

Age group (years)	Number	Total no.		Sex	Testing cohorts			Testing procedures		No. test sessions	
		Female no.	Male no.	Sig. diff of sex/age group	One	Two	Three	Online	Face-to-face	One session	Two sessions
5–5.11	17	8	9		0	0	17	17	0	4	13
6–6.11	18	8	10		0	0	18	18	0	8	10
7–7.11	23	13	10		0	10	13	13	10	20	3
8–8.11	18	10	8		0	9	9	9	9	17	1
9–9.11	17	9	8		5	8	4	4	13	17	0
10–10.11	27	12	15		15	7	5	5	22	27	0
11–11.11	17	8	9		4	6	7	7	10	17	0
12–12.11	18	10	8		0	6	12	12	6	18	0
13–13.11	16	8	8		0	2	14	14	2	16	0
	171	86	85	*p = 0.829*	24	48	99	99	72	144	27

Details of age in months and scores on standardized (general vocabulary) test measures and EVER (vocabulary of emotion) test measures have been tabulated and organized according to age group (see Table 2). Mean and standard deviations for females and males as well as overall for each age group have been calculated. There was a statistically significant difference between standard scores on the BPVS across age groups *F* (8, 162) = 2.045, *p* = 0.044. This was driven by higher mean scores in the 11–11.11 year group and relatively lower mean scores for the 6–6.11 year group. Group differences may have been affected by the use of BPVS version 2 or 3. However, we could not provide a corrected calculation of significance, due to the predominant usage of BPVS 2 in specific age groups (detailed in methods). For this reason, significant differences according to age group should be interpreted with caution. This difference according to age group does not affect our linear regression modeling or correlational analysis, but should be taken into account when interpreting counts of word items according to year group (see later in results section). For example, this type of skew in the data might result in a clustering of newly acquired word items at age 11–11.11 and a relatively lower number of newly acquired items at age 12–12.11. By contrast we might see fewer new items acquired at 6.6.11 years and relatively more at age 7–7.11. This will be reflected upon in the discussion.

**Table 2:** Showing total correct raw scores for BPVS, WA, WA sub-scores, and for The EVER Measure (WA:Emo and REV) and standard scores for BPVS.

Age group	Sex	Age in months *M*(SD)	BPVS RS *M*(SD) (max. 168)	BPVS SS *M*(SD)	WA Total RS *M*(SD)	WA Animals *M*(SD)	WA Food *M*(SD)	WA Jobs *M*(SD)	WA Emo *M*(SD)	REV Correct *M*(SD) (max. 39)	REV Close *M*(SD) (max. 39)	REV distant *M*(SD) (max. 39)
5–5.11	F	66.00 (4.66)	94.13 (7.32)	104.67 (3.72)	34.00 (5.93)	12.75 (3.24)	14.13 (4.91)	7.13 (1.46)	7.00 (2.20)	15.88 (7.14)	7.63 (2.50)	5.00 (3.96)
	M	67.22 (3.35)	94.56 (18.22)	104.67 (9.54)	26.78 (11.70)	11.78 (7.78)	10.00 (3.28)	5.00 (3.57)	6.00 (1.87)	14.22 (4.66)	7.56 (2.51)	6.33 (2.92)
Year group total		66.65 (3.94)	94.35 (13.77)	104.41 (7.19)	30.18 (9.88)	12.24 (5.92)	11.94 (4.52)	6.00 (2.92)	6.47 (2.04)	15.00 (5.82)	7.59 (2.43)	5.71 (3.41)
6–6.11	F	79.88 (4.05)	103.75 (17.19)	99.63 (11.36)	33.63 (10.32)	15.50 (6.14)	11.25 (2.38)	6.88 (2.70)	8.25 (2.17)	19.38 (7.35)	8.38 (2.56)	4.13 (2.42)
	M	75.70 (3.89)	99.90 (16.45)	101.00 (10.01)	34.20 (9.61)	14.80 (4.13)	11.90 (3.87)	7.50 (3.21)	5.80 (1.99)	18.20 (6.81)	8.00 (2.67)	5.40 (2.32)
Year group total		77.56 (4.40)	101.61 (16.39)	100.39 (10.33)	33.94 (9.64)	15.11 (4.97)	11.61 (3.22)	7.22 (2.92)	6.89 (2.59)	18.72 (6.87)	8.17 (2.55)	4.83 (2.28)
7–7.11	F	91.38 (2.66)	104.38 (20.90)	109.00 (8.32)	35.00 (8.20)	14.85 (3.36)	13.38 (4.07)	6.77 (2.59)	7.31 (2.14)	23.85 (5.24)	7.77 (2.86)	5.46 (2.11)
	M	88.40 (2.80)	102.00 (12.75)	102.20 (13.33)	37.00 (13.02)	14.50 (4.70)	14.50 (6.205)	8.00 (4.35)	5.80 (2.49)	20.20 (6.02)	7.90 (2.33)	5.60 (3.10)
Year group total		90.09 (3.06)	103.35 (17.50)	106.04 (11.06)	35.87 (10.35)	14.70 (3.90)	13.87 (5.01)	7.30 (3.43)	6.65 (2.37)	22.26 (5.76)	7.83 (2.59)	5.52 (2.52)
8–8.11	F	100.20 (3.23)	108.50 (23.18)	102.70 (8.18)	41.60 (8.92)	17.60 (3.86)	14.40 (3.92)	9.60 (3.06)	7.10 (2.42)	23.70 (6.96)	7.40 (3.75)	5.40 (2.84)
	M	98.75 (2.49)	105.50 (18.91)	102.50 (18.13)	38.38 (11.02)	15.13 (3.98)	13.75 (5.34)	9.50 (3.42)	10.00 (2.07)	22.38 (7.09)	7.50 (2.67)	4.63 (1.06)
Year group total		99.56 (2.94)	107.17 (20.83)	102.61 (13.07)	40.17 (9.74)	16.50 (4.00)	14.11 (4.47)	9.56 (3.13)	8.39 (2.66)	23.11 (6.84)	7.44 (3.22)	5.06 (2.21)
9–9.11	F	115.11 (3.33)	113.78 (18.01)	105.22 (10.28)	57.56 (8.29)	21.78 (5.65)	21.44 (2.79)	14.33 (3.08)	9.33 (2.60)	29.11 (2.98)	5.78 (2.17)	3.78 (2.11)
	M	113.75 (3.33)	113.88 (9.94)	113.88 (7.42)	50.63 (12.21)	18.50 (4.78)	19.00 (5.01)	13.13 (5.14)	10.25 (2.49)	26.13 (2.10)	7.50 (1.07)	5.38 (2.13)
Year group total		114.47 (3.30)	113.82 (14.33)	109.29 (9.84)	54.29 (10.60)	20.24 (5.37)	20.29 (4.06)	13.76 (4.09)	9.76 (2.51)	27.71 (2.95)	6.59 (1.91)	4.53 (2.21)
10–10.11	F	123.92 (3.83)	108.83 (10.58)	105.83 (9.59)	55.00 (10.49)	20.00 (4.05)	20.33 (5.07)	14.67 (3.26)	10.83 (3.83)	28.83 (4.49)	7.50 (3.50)	2.67 (2.19)
	M	126.27 (2.92)	121.73 (13.61)	112.60 (7.50)	54.73 (12.27)	20.07 (5.76)	20.07 (5.42)	14.60 (3.92)	10.13 (3.44)	26.80 (4.72)	8.20 (2.27)	3.20 (1.70)
Year group total		125.22 (3.49)	116.00 (13.78)	109.59 (9.00)	54.85 (11.30)	20.04 (4.98)	20.19 (5.17)	14.63 (3.58)	10.44 (3.57)	27.70 (4.65)	7.89 (2.85)	2.96 (1.91)
11–11.11	F	139.00 (2.93)	114.75 (14.70)	107.88 (15.84)	60.50 (11.35)	20.13 (4.58)	25.25 (5.15)	15.13 (3.31)	10.38 (2.77)	29.63 (4.81)	7.25 (2.71)	2.13 (2.36)
	M	137.56 (4.88)	142.44 (17.55)	115.56 (15.67)	59.67 (8.14)	22.56 (4.98)	22.33 (4.00)	14.78 (4.12)	10.33 (2.06)	30.89 (2.89)	5.11 (1.45)	3.00 (1.80)
Year group total		138.24 (4.02)	129.41 (21.25)	111.94 (15.75)	60.06 (9.47)	21.41 (4.81)	23.71 (4.67)	14.94 (3.65)	10.35 (2.34)	30.29 (3.84)	6.12 (2.34)	2.59 (2.06)
12–12.11	F	149.20 (4.02)	133.30 (15.33)	109.00 (11.61)	58.40 (3.13)	21.00 (2.75)	22.20 (3.33)	15.20 (3.65)	11.80 (3.52)	30.80 (3.16)	5.80 (2.20)	2.10 (0.88)
	M	150.38 (3.96)	142.63 (14.26)	106.75 (8.07)	65.38 (13.03)	23.38 (6.09)	23.63 (2.83)	18.38 (4.98)	12.38 (4.53)	31.13 (1.25)	5.75 (2.12)	2.13 (1.13)
Year group total		149.72 (3.92)	137.44 (15.20)	108.00 (9.98)	61.50 (9.37)	22.06 (4.56)	22.83 (3.11)	16.61 (4.46)	12.06 (3.89)	30.94 (2.44)	5.78 (2.10)	2.11 (0.96)
13–13.11	F	159.25 (2.66)	142.13 (13.25)	102.00 (10.09)	58.38 (12.45)	19.25 (7.94)	22.75 (5.20)	16.38 (1.85)	11.00 (3.96)	31.00 (3.51)	6.63 (3.58)	1.38 (0.52)
	M	161.50 (3.07)	148.25 (10.94)	106.25 (16.65)	64.63 (10.74)	23.50 (3.96)	24.38 (5.13)	16.75 (3.69)	10.88 (3.44)	30.25 (4.23)	5.63 (2.67)	3.00 (2.39)
Year group total		160.38 (3.01)	145.19 (12.16)	104.13 (13.48)	62.50 (11.69)	21.38 (6.45)	23.56 (5.06)	16.56 (2.83)	10.94 (3.59)	30.63 (3.78)	6.13 (3.10)	2.19 (1.87)

RS, Raw Scores; SS, Standard Scores; M, Mean; SD, Standard Deviation.

### Exploring proof of concept for The EVER Measure

**Figure 4:**
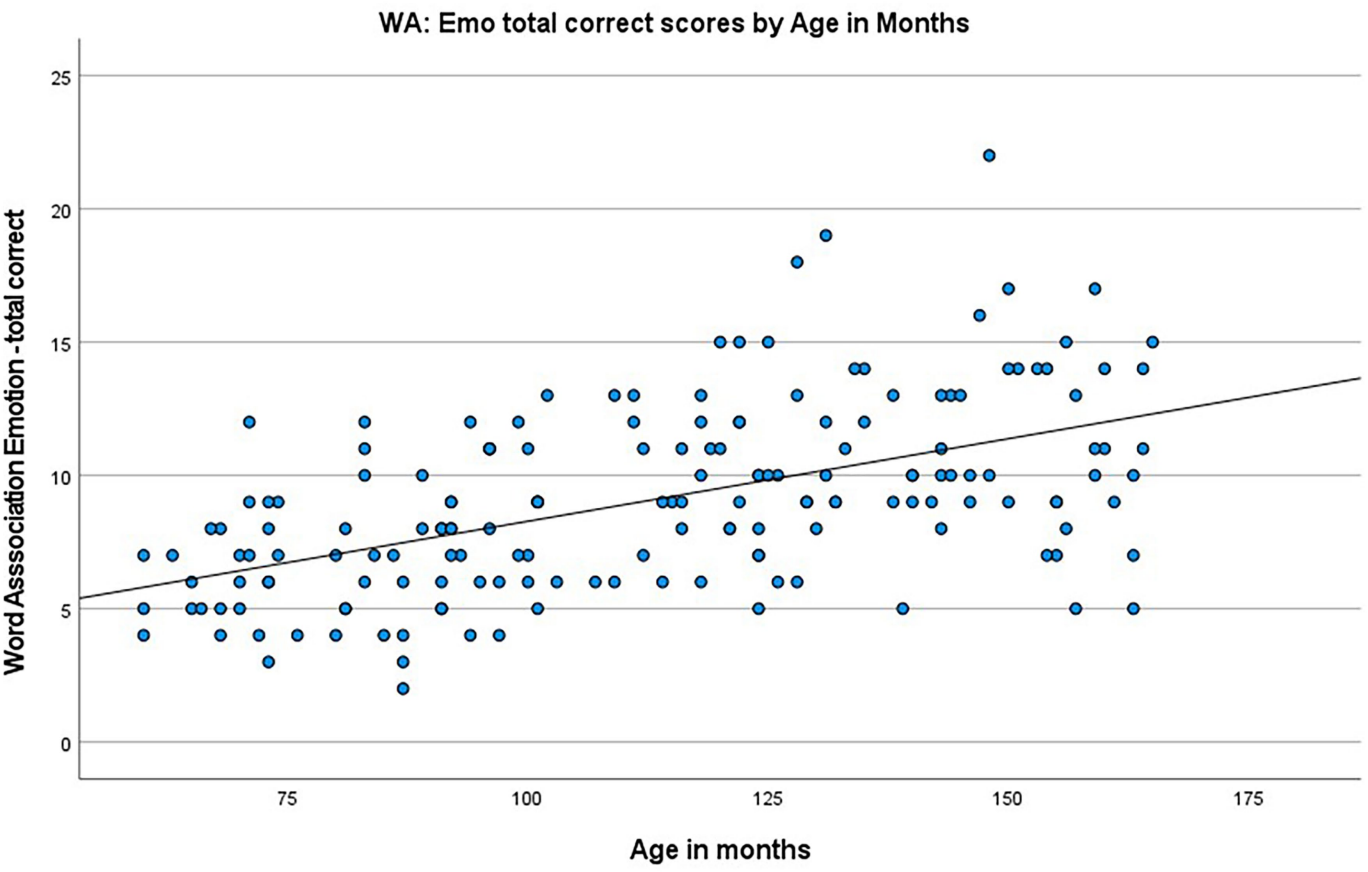
Scatterplot showing the correlation between WA:Emo scores and age in month.

*Assessing the acquisition of emotion vocabulary across childhood:* A multiple regression was used to identify possible predictors of scores on the Word Association Emotion Vocabulary (WA:Emo) task, with independent candidate variables, of age and sex and testing procedure. ‘Testing procedure’ was not hypothesized to affect the model, but was included at this stage to test for confounding influence. These variables predicted scores on the WA:Emo Task with statistical significance, *F*(3, 167) = 23.288, *p* < 0.001, *R^2^* = 0.295. Age in months (*B* = 0.060, *p* < 0.001) was significantly associated with WA:Emo Task, however sex (*B* = −0.081, *p* = 0.859) and testing procedure (*B* = −0.470, *p* = 0.142) were not. The relationship between age and WA:Emo score is depicted in Figure 4.

**Figure 5:**
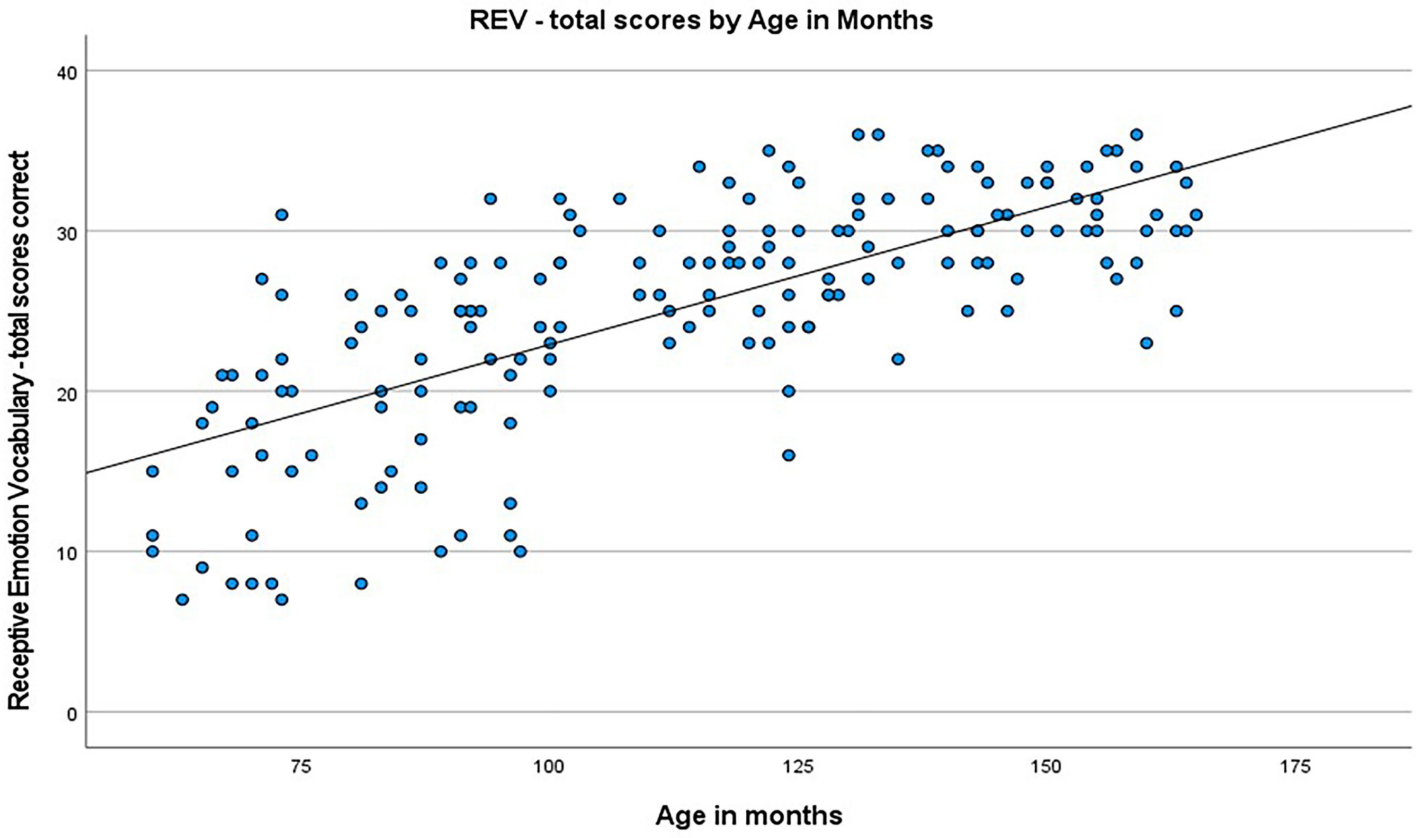
Scatterplot showing the correlation between REV scores and age in months.

Similarly, this process was applied to *total correct scores* on the Receptive Emotion Vocabulary (REV) task. These variables statistically significantly predicted scores on the WA:Emo Task, *F*(3, 167) = 61.955, *p* < 0.001, *R^2^* = 0.527. Age in months (*B* = 0.168, *p* < 0.001), was significantly associated with REV, however, sex (*B* = −1.303, *p* = 0.091) and testing procedure (*B* = −0.632, *p* = 0.243), were not. The relationship between age and REV score is depicted in Figure 5.

**Figure 6:**
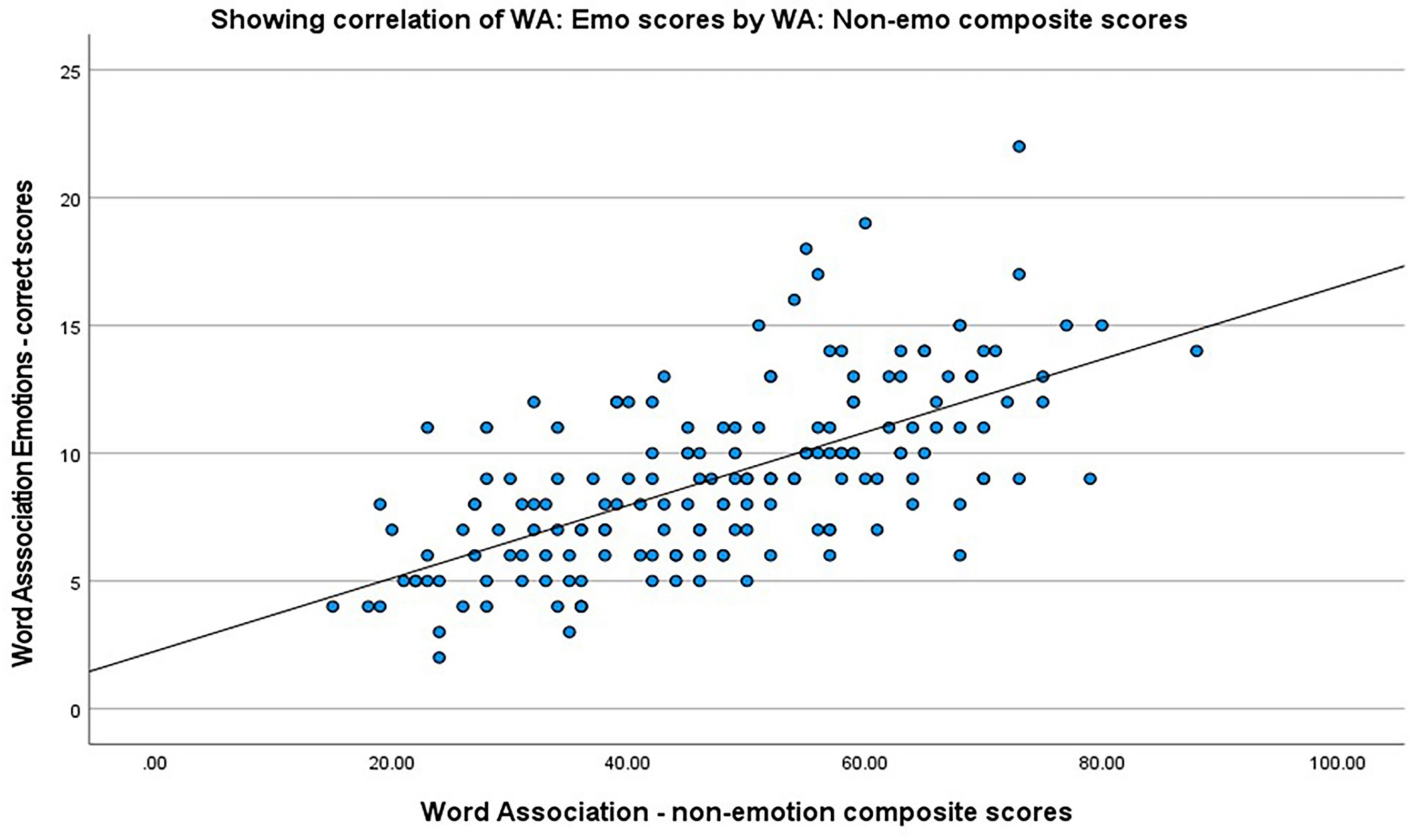
Scatterplot showing the correlation between WA:Emo and WA basic language composite scores.

*Correlation of EVER with standardized general vocabulary measures:* A Pearson bivariate correlation was run to determine the relationship between WA:Emo scores and WA basic language scores (which was a composite score of the subcategories: animals, food, occupations). As anticipated, there was a moderate, positive correlation between WA:Emo and WA basic language scores, which was statistically significant (*r* = 0.642, *n* = 171, *p* < 0.001) and can be seen in Figure 6.

**Figure 7:**
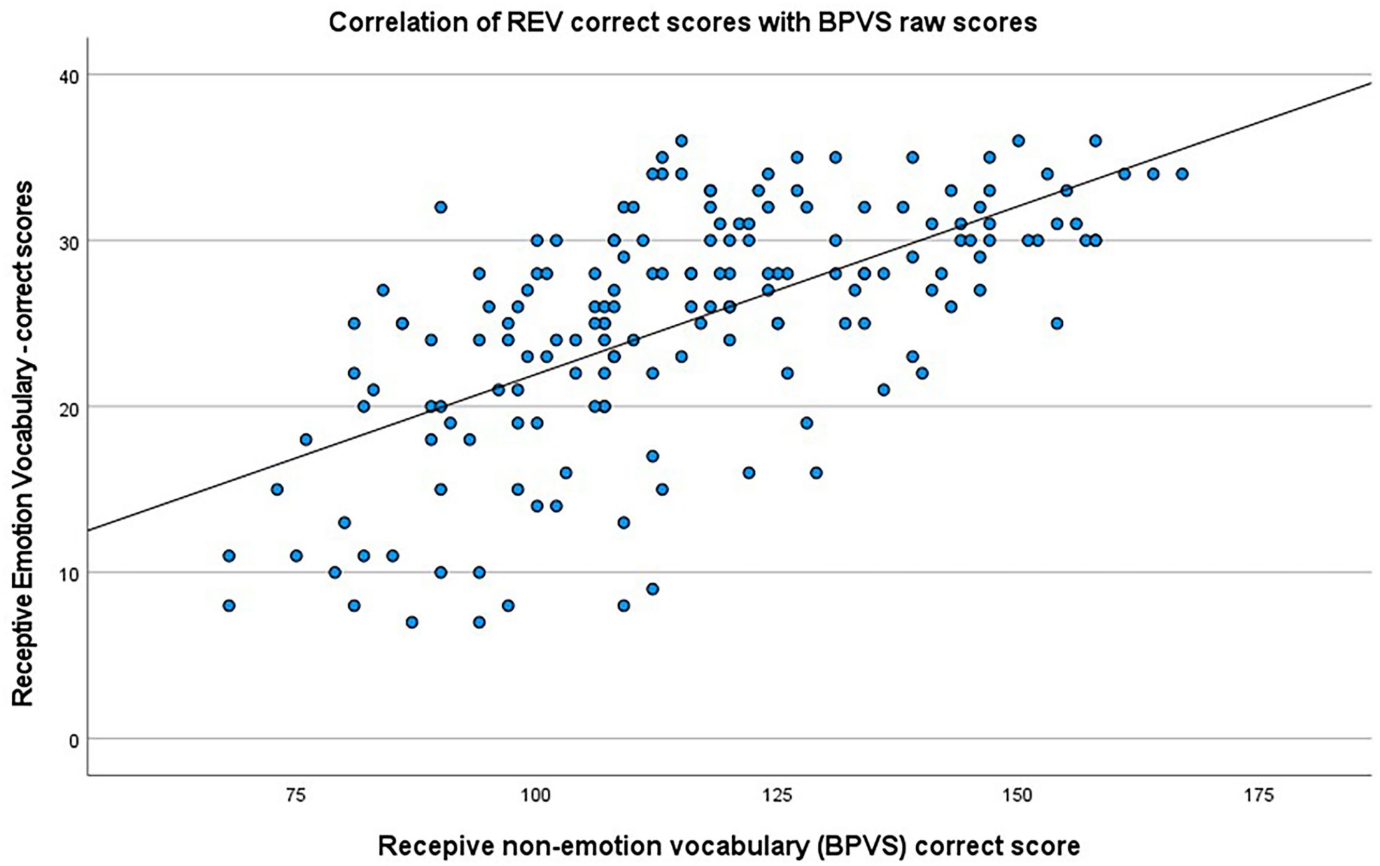
Scatterplot showing the correlation between REV scores and BPVS.

A correlation was run to determine the relationship between scores on the Receptive Emotion Vocabulary (REV) items and BPVS (raw scores). Again there was a moderate, positive correlation between REV and BPVS basic language scores, which was statistically significant (*r* = 0.632, *n* = 171, *p* < 0.001) and can be seen in Figure 7.

A partial correlation was then run to determine the relationship between scores on the Receptive Emotion Vocabulary (REV) items and BPVS (raw scores) after controlling for any difference incurred by using either BPVS version 2 and 3. There was a strong, positive partial correlation between REV correct score (*M*: 25.15, SD: 7.155) and BPVS raw score (*M*: 115.81, SD: 22.376) while controlling for BPVS version. This was statistically significant, *r* (168) = 0.761, *N* = 171, *p* < 0.001.

### Receptive Emotion Vocabulary: Age of acquisition of emotion vocabulary

An item by item analysis of the correct identification of receptive emotion vocabulary across age groups was conducted. All word items (*n* = 39) were tabulated in the order in which they occurred on the test stimuli (see Table 3). This included two occurrences of word stimuli ‘worried’ (order number 5 and 18) and ‘threatened’ (order number 12 and 26). The rationale for this inclusion is discussed above. Number of times an item was correctly identified in each age group and the percentage of children who correctly identified the item within each age group was calculated. In addition, the number of children choosing the close incorrect item was calculated alongside the percentage of that age group, e.g., 94.4% of 6-6.11 year olds got ‘surprised’ correct and 5.6% chose the close distractor.

**Table 3:** Showing REV responses organized by word item and age.

REV word item		Age 5–5.11 *n* = 17	Age 6–6.11 *n* = 18	Age 7–7.11 *n* = 23	Age 8–8.11 *n* = 18	Age 9–9.11 *n* = 17	Age 10–10.11 *n* = 27	Age 11–11.11 *n* = 17	Age 12–12.11 *n* = 18	Age 13–13.11 *n* = 16
1. Happy	Correct (%)	**17 (100)**	18 (100)	23 (100)	18 (100)	17 (100)	26 (96.3)	17 (100)	17 (94.4)	16 (100)
	Close (%)	0	0	0	0	0	1 (3.7)	0	1 (5.6)	0
2. Sad	Correct (%)	7 (41.2)	7 (38.9)	15 (65.2)	**13 (72.2)**	8 (47.1)	19 (70.4)	12 (70.6)	14 (77.8)	11 (68.8)
	Close (%)	10 (58.8)	11 (61.1)	8 (34.8)	5 (27.8)	9 (52.9)	8 (29.6)	5 (29.4)	4 (22.2)	5 (31.3)
3. Afraid	Correct (%)	**16 (94.1)**	18 (100)	21 (91.3)	18 (100)	17 (100)	27 (100)	16 (94.1)	18 (100)	16 (100)
	Close (%)	1 (5.9)	0	2 (8.7)	0	0	0	1 (5.9)	0	0
4. Excited	Correct (%)	**13 (76.5)**	18 (100)	22 (95.7)	17 (94.4)	17 (100)	25 (92.6)	16 (94.1)	18 (100)	16 (100)
	Close (%)	4 (23.5)	0	1 (4.3)	1 (5.6)	0	2 (7.4)	1 (5.9)	0	0
5. Worried_1	Correct (%)	**16 (94.1)**	16 (88.9)	21 (91.3)	16 (88.9)	17 (100)	24 (88.9)	17 (100)	17 (4.4)	14 (87.5)
	Close (%)	1 (5.9)	2 (11.1)	2 (8.7)	2 (11.1)	0	2 (7.4)	0	1 (5.6)	2 (12.5)
6. Interested	Correct (%)	11 (64.7)	11 (61.1)	16 (69.6)	9 (50.0)	**13 (76.5)**	17 (63.0)	15 (88.2)	18 (100)	13 (81.3)
	Close (%)	5 (29.4)	6 (33.3)	6 (26.1)	9 (50.0)	4 (23.5)	10 (37.0)	2 (11.18)	0	3 (18.8)
7. Angry	Correct (%)	**14 (82.4)**	18 (100)	21 (91.3)	18 (100)	17 (100)	27 (100)	16 (94.1)	17 (94.4)	15 (93.8)
	Close (%)	3 (17.6)	0	2 (8.7)	0	0	0	1 (5.9)	1 (5.6)	1 (6.3)
8. Surprised	Correct (%)	10 (58.8)	**17 (94.4)**	20 (87.0)	12 (66.7)	15 (88.2)	22 (81.5)	13 (76.5)	15 (83.3)	16 (100)
	Close (%)	7 (41.2)	1 (5.6)	3 (13.0)	6 (33.3)	2 (11.8)	2 (7.4)	1 (5.9)	1 (5.6)	0
9. Disgusted	Correct (%)	**16 (94.1)**	18 (100)	21 (91.3)	18 (100)	16 (94.1)	27 (100)	17 (100)	18 (100)	15 (93.8)
	Close (%)	1 (5.9)	0	2 (8.7)	0	1 (5.9)	0	0	0	1 (6.3)
10. Confused	Correct (%)	10 (58.8)	**14 (77.8)**	17 (73.9)	14 (77.8)	16 (94.1)	23 (85.2)	11 (64.7)	13 (72.2)	13 (81.3)
	Close (%)	5 (29.4)	2 (11.1)	6 (26.1)	4 (22.2)	1 (5.9)	4 (14.8)	3 (17.6)	5 (27.8)	3 (18.8)
11. Calm	Correct (%)	11(64.7)	11 (61.1)	**17 (73.9)**	13 (72.2)	12 (70.6)	23 (85.2)	16 (94.1)	16 (88.9)	12 (75.0)
	Close (%)	6 (35.3)	6 (33.3)	6 (26.1)	5 (27.8)	5 (29.4)	4 (14.8)	1 (5.9)	2 (11.1)	4 (25.0)
12. Threatened_1	Correct (%)	7 (41.2)	8 (44.4)	9 (39.1)	**13 (72.2)**	11 (64.7)	12 (44.4)	10 (58.8)	13 (72.2)	10 (62.5)
	Close (%)	5 (29.4)	6 (33.3)	8 (34.8)	4 (22.2)	3 (17.6)	10 (37.0)	6 (35.3)	3 (16.7)	5 (31.3)
13. Proud	Correct (%)	**15 (88.2)**	1 (5.6)	4 (17.4)	6 (33.3)	11 (64.7)	13 (48.1)	10 (58.8)	8 (44.4)	9 (56.3)
	Close (%)	1 (5.9)	17 (94.4)	19 (82.6)	12 (66.7)	6 (35.3)	14 (51.9)	7 (41.2)	10 (55.6)	7 (43.8)
14. Embarrassed	Correct (%)	4 (23.5)	7 (38.9)	6 (26.1)	9 (50.0)	8 (47.1)	13 (48.1)	10 (58.8)	11 (61.1)	6 (37.5)
	Close (%)	10 (58.8)	8 (44.4)	10 (43.5)	5 (27.8)	4 (23.5)	5 (18.5)	4 (23.5)	3 (16.7)	7 (43.8)
15. Thoughtful	Correct (%)	8 (47.1)	9 (50.0)	10 (43.5)	12 (66.7)	11 (64.7)	18 (66.7)	**13 (76.5)**	14 (77.8)	14 (87.5)
	Close (%)	7 (41.2)	1 (5.6)	1 (4.3)	6 (33.3)	6 (35.3)	9 (33.3)	1 (5.9)	4 (22.2)	2 (12.5)
16. Overjoyed	Correct (%)	8 (47.1)	11 (61.1)	**19 (82.6)**	13 (72.2)	16 (94.1)	21 (77.8)	15 (88.2)	16 (88.9)	15 (93.8)
	Close (%)	4 (23.5)	4 (22.2)	3 (13.0)	2 (11.1)	1 (5.9)	4 (14.8)	2 (11.8)	2 (11.1)	1 (6.3)
17. Guilty	Correct (%)	7 (41.2)	9 (50.0)	13 (56.5)	12 (66.7)	11 (64.7)	16 (59.3)	**14 (82.4)**	13 (72.2)	10 (62.5)
	Close (%)	2 (11.8)	5 (27.8)	7 (30.4)	4 (22.2)	4 (23.5)	11 (40.7)	2 (11.8)	5 (27.8)	5 (31.3)
18. Worried_2	Correct (%)	9 (52.9)	12 (66.7)	**20 (87.0)**	17 (94.4)	14 (82.4)	24 (88.9)	15 (88.2)	18 (100)	12 (75.0)
	Close (%)	3 (17.6)	3 (16.7)	1 (4.3)	1 (5.6)	3 (17.6)	3 (11.1)	2 (11.8)	0	4 (25.0)
19. Amused	Correct (%)	1 (5.9)	2 (11.1)	7 (30.4)	6 (33.3)	11 (64.7)	16 (59.3)	**13 (76.5)**	17 (94.4)	15 (93.8)
	Close (%)	1 (5.9)	4 (22.2)	4 (17.4)	5 (27.8)	3 (17.6)	9 (33.3)	3 (17.6)	1 (5.6)	1 (6.3)
20. Startled	Correct (%)	9 (52.9)	8 (44.4)	**17 (73.9)**	13 (72.2)	17 (100)	24 (88.9)	16 (94.1)	16 (88.9)	12 (75.0)
	Close (%)	1 (5.9)	3 (16.7)	4 (17.4)	4 (22.2)	0	3 (11.1)	1 (5.9)	2 (11.1)	4 (25.0)
21. Frustrated	Correct (%)	4 (23.5)	5 (27.8)	8 (34.8)	4 (22.2)	7 (41.2)	13 (48.1)	9 (52.9)	10 (55.6)	6 (37.5)
	Close (%)	5 (29.4)	10 (55.6)	12 (52.2)	13 (72.2)	10 (58.8)	14 (51.9)	8 (47.1)	7 (38.9)	10 (62.5)
22. Affectionate	Correct (%)	3 (17.6)	3 (16.7)	7 (30.4)	7 (38.9)	7 (41.2)	**19 (70.4)**	14 (82.4)	18 (100)	14 (87.5)
	Close (%)	3 (17.6)	10 (55.6)	3 (13.0)	1 (5.6)	5 (29.4)	5 (18.5)	2 (11.8)	0	1 (6.3)
23. Gloomy	Correct (%)	8 (47.1)	**13 (72.2)**	18 (78.3)	15 (83.3)	15 (88.2)	26 (96.3)	15 (88.2)	18 (100)	15 (93.8)
	Close (%)	3 (17.9)	1 (5.6)	3 (13.0)	1 (5.6)	1 (5.9)	1 (3.7)	1 (5.9)	0	1 (6.3)
24. Enthusiastic	Correct (%)	2 (11.8)	6 (33.3)	14 (60.9)	12 (66.7)	**15 (88.2)**	21 (77.8)	14 (82.4)	16 (88.9)	15 (93.8)
	Close (%)	1 (5.9)	5 (27.8)	2 (8.7)	1 (5.6)	2 (11.8)	6 (22.2)	3 (17.6)	2 (11.1)	1 (6.3)
25. Stubborn	Correct (%)	1 (5.9)	4 (22.2)	9 (39.1)	11 (61.1)	10 (58.8)	18 (66.7)	11 (64.7)	**15 (83.3)**	15 (93.8)
	Close (%)	7 (41.2)	6 (33.3)	8 (34.8)	4 (22.2)	5 (29.4)	7 (25.9)	5 (29.4)	3 (16.7)	1 (6.3)
26. Threatened_2	Correct (%)	7 (41.2)	3 (16.7)	14 (60.9)	4 (22.2)	11 (64.7)	17 (63.0)	11 (64.7)	**15 (83.3)**	14 (87.5)
	Close (%)	2 (11.8)	2 (11.1)	2 (8.7)	5 (27.8)	4 (23.5)	7 (25.9)	3 (17.6)	1 (5.6)	1 (6.3)
27. Distant	Correct (%)	1 (5.9)	4 (22.2)	10 (43.5)	6 (33.3)	**12 (70.6)**	19 (70.4)	13 (76.5)	17 (94.4)	13 (81.3)
	Close (%)	9 (52.9)	2 (11.1)	2 (8.7)	3 (16.7)	5 (29.4)	3 (11.1)	1 (5.9)	1 (5.6)	1 (6.3)
28. Determined	Correct (%)	3 (17.9)	7 (38.9)	5 (21.7)	8 (44.4)	9 (52.9)	14 (51.9)	**12 (70.6)**	15 (83.3)	13 (81.3)
	Close (%)	5 (29.4)	3 (16.7)	3 (13.0)	1 (5.6)	2 (11.8)	5 (18.5)	2 (11.8)	3 (16.7)	3 (18.8)
29. Devastated	Correct (%)	1 (11.8)	6 (33.3)	13 (56.5)	8 (44.4)	**16 (94.1)**	25 (92.6)	16 (94.1)	18 (100)	16 (100)
	Close (%)	6 (35.3)	5 (27.8)	5 (21.7)	5 (27.8)	1 (5.9)	2 (7.4)	1 (5.9)	0	0
30. Adoring	Correct (%)	4 (23.5)	10 (55.6)	9 (39.1)	11 (61.1)	**14 (82.4)**	19 (70.4)	14 (82.4)	16 (88.9)	9 (56.3)
	Close (%)	1 (5.9)	1 (5.6)	9 (39.1)	2 (11.1)	3 (17.6)	8 (29.6)	3 (17.6)	2 (11.1)	7 (43.8)
31. Baffled	Correct (%)	1 (5.9)	4 (22.2)	10 (43.5)	6 (33.3)	7 (41.2)	10 (37.0)	**12 (70.6)**	11 (61.1)	4 (25.0)
	Close (%)	4 (23.5)	4 (22.2)	5 (21.7)	3 (16.7)	2 (11.8)	13 (48.1)	3 (17.6)	5 (27.8)	6 (37.5)
32. Humiliated	Correct (%)	3 (17.6)	6 (33.3)	10 (43.5)	8 (44.4)	8 (47.1)	10 (37.0)	7 (41.2)	8 (44.4)	10 (62.5)
	Close (%)	3 (17.6)	4 (22.2)	6 (26.1)	5 (27.8)	8 (47.1)	16 (59.3)	9 (52.9)	10 (55.6)	6 (37.5)
33. Contemptuous	Correct (%)	2 (11.8)	4 (22.2)	13 (56.5)	9 (50.0)	8 (47.1)	15 (55.6)	**12 (70.6)**	9 (50.0)	14 (87.5)
	Close (%)	5 (29.4)	5 (27.8)	3 (13.0)	4 (22.2)	6 (35.3)	9 (33.3)	3 (17.6)	3 (16.7)	1 (6.3)
34. Choosing	Correct (%)	6 (35.3)	9 (50.0)	13 (56.5)	8 (44.4)	**12 (70.6)**	19 (70.4)	15 (88.2)	8 (44.4)	10 (62.5)
	Close (%)	0	1 (5.6)	4 (17.4)	5 (27.8)	4 (23.5)	7 (25.9)	2 (11.8)	8 (44.4)	4 (25.0)
35. Intimidated	Correct (%)	2 (11.8)	2 (11.1)	9 (39.1)	4 (22.2)	9 (52.9)	**20 (74.1)**	12 (70.6)	12 (66.7)	13 (81.3)
	Close (%)	3 (17.6)	8 (44.4)	6 (26.1)	4 (22.2)	5 (29.4)	3 (11.1)	3 (17.6)	3 (16.7)	1 (6.3)
36. Mystified	Correct (%)	1 (5.9)	3 (16.7)	6 (26.1)	9 (50.0)	11 (64.7)	**19 (70.4)**	13 (76.5)	12 (66.7)	10 (62.5)
	Close (%)	2 (11.8)	4 (22.2)	5 (21.7)	1 (5.6)	3 (17.6)	2 (7.4)	4 (23.5)	3 (16.7)	5 (31.3)
37. Empathic	Correct (%)	4 (23.5)	4 (22.2)	7 (30.4)	2 (11.1)	6 (35.3)	14 (51.9)	9 (52.9)	**13 (72.2)**	14 (87.5)
	Close (%)	1 (5.9)	2 (11.1)	3 (13.0)	2 (11.1)	1 (5.9)	3 (11.1)	5 (29.4)	3 (16.7)	1 (6.3)
38. Indifferent	Correct (%)	3 (17.6)	3 (16.7)	5 (21.7)	7 (38.9)	5 (29.4)	12 (44.4)	8 (47.1)	10 (55.6)	**12 (75.0)**
	Close (%)	3 (17.6)	2 (11.1)	8 (34.8)	4 (22.2)	7 (41.2)	9 (33.3)	7 (41.2)	6 (33.3)	3 (18.8)
39. Stern	Correct (%)	3 (17.6)	7 (38.9)	13 (56.5)	10 (55.6)	**14 (82.4)**	21 (77.8)	16 (94.1)	10 (55.6)	13 (81.3)
	Close (%)	2 (11.8)	2 (11.1)	1 (4.3)	1 (5.6)	2 (11.8)	4 (14.8)	1 (5.9)	7 (38.9)	2 (12.5)

Number of correct responses (and percentage), and close incorrect responses (and percentage) for each age group are provided. Highlighted and emboldened items represent the earliest age at which a word is correctly identified by > 70% of children.

Items that were identified correctly by at least 70% of the child participants in any age group were noted. They are highlighted in the table for the youngest age group in which they meet this threshold. For example, ‘surprised’ is correctly identified by 94.4% of the children (aged 6–6.11) and remains correctly identified across the older age groups. However, it is only highlighted on the first instance. Thirty six words met the threshold of ≥ 70% in at least one age group. *N* = 7 words (happy, afraid, excited, worried_1, angry, disgusted, proud) were correctly identified by ≥ 70% of the group for the first time at 5–5.11 years; *N* = 3 words (surprised, confused, gloomy) at age 6–6.11; *N* = 4 words (calm, overjoyed, worried_2, startled) at 7–7.11; *N* = 2 words (sad and threatened) at 8–8.11; *N* = 7 words (interested, enthusiastic, distant, devastated, adoring, choosing, stern) at 9–9.11; *N* = 3 words (affectionate, intimidated, mystified) at 10–10.11; *N* = 6 words (thoughtful, guilty, amused, determined, baffled, contemptuous) at 11–11.11; *N* = 3 words (stubborn, threatened_2, empathic) at 12–12.11; *N* = 1 word (indifferent) 13–13.11. *N* = 3 words were never identified correctly by over 70% of children (humiliated, frustrated, embarrassed).

### Age of emergence of word items using the Word Association: Emotions task

All correct word items (*n* = 225) were organized and tabulated in alphabetical order (see Table 4). The following words were the most commonly produced, with the percentage denoting the amount of children who generated that response: happy (90.64%), sad (89.47%), embarrassed (66.67%), angry (66.01%), excited (44.44%), scared (34.50%), annoyed (23.98%), upset (20.47%), worried (18.71%), nervous (17.54%), confused (16.37%), joyful (15.21%), shocked (15.21%). In Table 4, each age group is represented by a column that shows the total number of occurrences of each word and the percentage of children generating that word in that age group. For example, angry was generated by 51% of 5 year olds. The total number of times each word was generated and the percentage of all children generating that word across all age groups is in the final (right-hand) column. The total number of words generated by each age group can be found in the ultimate row of the table. While there is no precedent for establishing a threshold of emergence in this type of data, the current study operationalized emergence as ≥ 10% of responses in any age group. A high threshold (as in the REV task) would not expected in a word association task where children would not necessarily generate all words known in category.’ This threshold was selected based on a perusal of the tabulated dataset. Considerations in the selection process were to establish a threshold low enough so that it did not ‘wash out’ important trends in the data by missing emerging words. For example, very few words reached 20% significance and 15% resulted in a skew toward a later age of emergence for many words which might be considered earlier emerging. Secondly, it was not so low as to over-represent insignificant occurrence of words. In adopting the ≥ 10% threshold the data showed a spread of results with a selection of word items emerging for the first time across all age brackets (as might be expected). In addition, it is generally the case that expressive words would emerge subsequent to receptive understanding of the same word (Hendriks and Koster, 2010). This was the case with nearly all word items where a direct comparison could be made, either with the REV measure or the findings from Baron-Cohen et al. (2010). Exceptions to this are considered in the discussion.

**Table 4:** Showing all correct word items generated in the WA:Emo task (1) total number of occurrences for each word per age group, (2) percentage of children generating each word in each age group, (3) total number of times each word was generated across all ages, (4) percentage of all children generating that word, (5) total number of words generated by each age group, (6) average number of words generated by females and males per age group.

No	Word item	5–5.11 *n* = 17		6–6.11 *n* = 18		7–7.11 *n* = 23		8–8.11 *n* = 18		9–9.11 *n* = 17		10–10.11 *n* = 27		11–11.11 *n* = 17		12–12.11 *n* = 18		13–13.11 *n* = 16		All ages	
		*n*	%	*n*	%	*n*	%	*n*	%	*n*	%	*n*	%	*n*	%	*n*	%	*n*	%	Total	%
1	Adventurous											1	1							1	0.585
2	Afraid					1	1	1	5.6			1	1	1	1					4	2.339
3	Aggravated									1	5.9					2	**13**			3	1.754
4	Aggressive													3	7.9	1	6.3			4	2.339
5	Amazed			1	1	1	4.3	1	1			1	3.4	1	1	2	**13**			7	4.094
6	Amazing									1	5.9									1	0.585
7	Amused									1	5.9					1	1			2	1.170
8	Anger									1	5.9	4	**11**			2	2	1	6.3	8	4.678
9	Angry	12	**51**	14	46	16	33	11	38	11	35	13	33	12	36	12	49	12	59	113	66.082
10	Annoyed			3	3	3	6.3	4	**13**	6	26	6	16	7	27	8	34	4	20	41	23.977
11	Anxious			3	3	1	1				0	2	4.4	3	3	3	3	6	**17**	18	10.526
12	Apprehensive																	1	6.3	1	0.585
13	Ashamed					2	2													2	1.170
14	Astonished									1	1			1	5.9					2	1.170
15	Awful							1	5.6											1	0.585
16	Awkward													1	5.9					1	0.585
17	Bad					1	1			1	5.9							1	1	3	1.754
18	Baffled											1	1							1	0.585
19	Bemused													1	5.9					1	0.585
20	Bewildered											1	1							1	0.585
21	Blue			1	5.6															1	0.585
22	Bored	2	6.9	2	6.6	2	2	1	5.6	2	**12**	4	11	1	5.9	2	13	3	14	19	11.111
23	Brave	1	1			1	1	1	1							1	1			4	2.339
24	Calm	1	5.9							1	5.9	1	1					2	**13**	5	2.924
25	Caring					1	1					1	1							2	1.170
26	Cautious													2	**12**	1	6.3			3	1.754
27	Cheerful	1	5.9			1	1					1	3.4	1	1	3	**14**			7	4.094
28	Cold											1	3.4							1	0.585
29	Collected									1	5.9									1	0.585
30	Comfortable															1	6.3			1	0.585
31	Concerned																	1	6.3	1	0.585
32	Confident													1	5.9	1	6.3	2	2	4	2.339
33	Confused	2	2	3	**12**	1	4.3	2	6.6	1	1	5	7.4	3	13	3	8.3	8	29	28	16.374
34	Content											1	3.4	1	5.9			1	6.3	3	1.754
35	Cool									1	5.9									1	0.585
36	Cross	3	3	2	6.6	2	5.3	1	1			2	6.9			1	1			11	6.433
37	Cruel							1	5.6											1	0.585
38	Curious									2	**12**	1	1					1	1	4	2.339
39	Delighted															1	6.3			1	0.585
40	Depressed			1	1			1	5.6			3	**10**	2	2	5	16	7	23	19	11.111
41	Depression															12	**12**			12	7.018
42	Deserted													1	1					1	0.585
43	Despairing											1	1							1	0.585
44	Despise													1	5.9					1	0.585
45	Determination											1	3.4							1	0.585
46	Determined							1	5.6					1	5.9					2	1.170
47	Devastated																	1	6.3	1	0.585
48	Devoted													3				1	6.3	1	0.585
49	Disappointed	1	1					3	**17**			3	7.9	4	8.9					11	6.433
50	Discombobulated													1	1					1	0.585
51	Disgust							1	1			2	4.4			1	1	1	1	5	2.924
53	Disgusted			1	1			2	6.6			4	8.9			2	**13**	3	14	12	7.018
54	Dishonest											1	3.4							1	0.585
55	Dismay													1	1					1	0.585
56	Distain													1	5.9					1	0.585
57	Distracted											2	6.9							2	1.170
58	Distraught											1	1			1	1	2	**13**	4	2.339
59	Distressed									1	1	2	2							3	1.754
60	Dizzy											1	3.4							1	0.585
61	Down											2	4.4							2	1.170
62	Eager															1	1			1	0.585
63	Ecstatic											1	1	2	6.9			1	1	4	2.339
64	Elated											1	3.4			1	6.3			2	1.170
65	Embarrassed	10	**34**	12	35	13	33	11	38	14	43	19	41	11	45	11	48	13	50	114	66.667
66	Embarrassment											1	3.4			1	1			2	1.170
67	Emotional															2	2	1	1	3	1.754
68	Empathic															1	6.3			1	0.585
69	Empty													1	1					1	0.585
70	Energetic													1	5.9	1	6.3	1	6.3	3	1.754
71	Energized									1	1							1	1	2	1.170
72	Enjoyment													1	1					1	0.585
73	Enraged											1	3.4					1	6.3	1	0.585
74	Enthusiastic							1	1			2	2	2	6.9			1	6.3	6	3.509
75	Envious											1	3.4			1	1			2	1.170
76	Envy									1	1			1	5.9					2	1.170
78	Exasperated															1	1			1	0.585
79	Excited	5	**15**	9	32	5	12	15	42	7	17	11	28	9	29	6	17	9	25	76	44.444
80	excitement											1	3.4							1	0.585
81	exhausted					1	1									2	7.3	1	1	4	2.339
82	exhilarated																	1	1	1	0.585
83	fear							1	5.6			7	**19**			2	2			10	5.848
84	Fearful							1	1			1	3.4	1	5.9	2	7.3	2	7.3	7	4.094
85	fearless							2	6.6											2	1.170
86	Festive											1	3.4							1	0.585
87	Fine							1	5.6											1	0.585
88	Flabbergasted			1	5.6															1	0.585
89	Friendly															2	7.3			2	1.170
90	Frightened					1	1	1	5.6			1	3.4			1	1	1	1	5	2.924
91	Frustrated	3	7.9	1	1			1	5.6			1	3.4	2	2	3	8.3			11	6.433
92	Frustration									1	5.9									1	0.585
93	Furious			1	1	1	4.3			1	5.9	1	3.4					2	7.3	6	3.509
94	Generous															1	1			1	0.585
95	Glad							1	5.6											1	0.585
96	Gloomy					1	1			1	5.9	1	3.4							3	1.754
97	Good																	1	1	1	0.585
98	Grateful					1	1					1	1							2	1.170
99	Greedy							1	5.6							1	1			2	1.170
101	Grumpy	3	7.9							5	**20**	5	12	2	12	1	6.3	1	1	17	9.942
102	Guilty							1	5.6	1	5.9									2	1.170
103	Happiness															2	7.3			2	1.170
104	Happy	18	**67**	18	64	16	39	17	49	17	61	22	51	14	58	14	51	19	72	155	90.643
105	Hassled											1	1							1	0.585
106	Hate											1	3.4	2	**12**					3	1.754
107	Hatred															1	1	1	6.3	2	1.170
108	Heartbroken																	1	1	1	0.585
109	Honest											1	3.4							1	0.585
110	Hope											1	3.4							1	0.585
111	Hopefully			1	5.6															1	0.585
112	Humble															1	6.3			1	0.585
113	Humiliated									1	1			1	1	1	1			3	1.754
114	Hungry	3	**13**			1	1	1	5.6	1	1					1	1			7	4.094
115	Hurried											1	1							1	0.585
116	Hurt	1	5.9									1	3.4	1	5.9	1	6.3			4	2.339
118	Innocent									1	5.9									1	0.585
119	Inspired											1	3.4							1	0.585
120	Interested					1	1					1	1			1	1	2	7.3	5	2.924
121	Intimidated											2	4.4							2	1.170
122	Irritated															1	6.3	2	7.3	3	1.754
123	Isolated									1	5.9	1	3.4	1	1	1	6.3			4	2.339
124	Jealous					3	6.3			2	2	2	6.9	2	**12**	3	8.3	1	1	13	7.602
125	Jealousy															1	6.3			1	0.585
126	Jolly							1	5.6							1	1			2	1.170
127	Joy			1	1					1	5.9	3	7.9			1	1	1	1	7	4.094
128	Joyful							3	7.6	4	**14**	8	13	4	14	3	8.3	4	9.3	26	15.205
129	Joyous											1	1			3	**19**	1	6.3	5	2.924
130	Kind					1	4.3									1	1			2	1.170
131	Let down							1	5.6											1	0.585
132	Lonely					5	**15**	2	11	1	5.9	2	4.4	4	8.9	3	8.3	1	1	18	10.526
133	Love	1	5.9			1	1			1	1	1	3.4	2	**12**			1	1	7	4.094
134	Loving															1	6.3			1	0.585
135	Lucky							1	5.6											1	0.585
136	Mad			1	1	2	8.7	2	6.6	1	1	2	6.9	1	5.9			2	2	11	6.433
137	Malicious																	1	6.3	1	0.585
138	Mean											2	6.9							2	1.170
139	Melancholy													1	1			1	6.3	2	1.170
140	Miserable	1	1			1	4.3					1	1			1	1			4	2.339
141	Misunderstood									1	5.9									1	0.585
142	Moaning					1	1													1	0.585
143	Moody											1	3.4	2	2	1	1			4	2.339
144	Mortified													1	1					1	0.585
145	Nasty							1	1							1	1			2	1.170
146	Nervous	3	7.9	2	2	4	7.3	4	8.6	3	**13**	3	5.4	5	15	4	9.3	2	2	30	17.544
147	Obnoxious									1	1									1	0.585
148	Offended															1	6.3			1	0.585
149	Okay							2	**11**											2	1.170
150	Outraged			1	5.6									1	1					2	1.170
151	Over excited													1	1					1	0.585
152	Over the moon											1	3.4					1	6.3	2	1.170
153	Overjoyed								1	2	6.9	1	3.4	1	5.9	3	3			8	4.678
154	Overwhelmed													1	1	4	**20**			5	2.924
155	Pain															1	1	1	1	2	1.170
156	Panicking											1	1							1	0.585
157	Passionate															1	6.3			1	0.585
158	Peaceful											1	1							1	0.585
159	Perplexed											1	3.4					1	6.3	2	1.170
160	Petrified	2	**12**	1	5.6							3	5.4			1	1			7	4.094
161	Playful			1	1	1	1			1	1									3	1.754
162	Pleasant											1	1							1	0.585
163	Pleased	1	1																	1	0.585
164	Positive													1	1					1	0.585
165	Prepared									1	1									1	0.585
166	Pressured															1	6.3			1	0.585
167	Proud					2	2	1	5.6	1	5.9	3	7.9			1	1			8	4.678
168	Puzzled											2	4.4							2	1.170
169	Rage																	1	6.3	1	0.585
170	Rejected															1	1			1	0.585
171	Relaxed									1	1	1	3.4			1	6.3			3	1.754
172	Relief							1	5.6											1	0.585
173	Reluctant											1	3.4							1	0.585
174	Sad	13	**52**	17	58	23	56	17	49	16	55	23	55	17	61	13	45	12	46	153	89.474
175	Sadly			1	5.6															1	0.585
176	Sadness											2	6.9			3	8.3	1	6.3	6	3.509
177	Satisfied													1	5.9					1	0.585
178	Scared	7	**22**	8	35	5	12	7	16	7	22	12	22	3	7.9	2	7.3	8	24	59	34.503
179	self-conscious																	1	1	1	0.585
180	Selfish											1	1			1	1			2	1.170
181	Selfless											1	1			1	1			2	1.170
182	Serene																	1	6.3	1	0.585
183	Serious					1	1							1	1	1	1			3	1.754
184	Shaky																	1	6.3	1	0.585
185	Shock											1	1							1	0.585
186	Shocked	2	2	3	7.6	4	**14**	3	7.6	3	3	5	9.9	5	15			1	1	26	15.205
187	Shy			1	1	3	6.3	2	6.6	2	6.9	2	4.4			1	1	1	1	12	7.018
188	Sick	1	1	2	6.6			1	5.6	1	5.9	3	7.9	2	**12**					10	5.848
189	Silly	1	1					2	2			2	2	1	5.9					6	3.509
190	Sleepiness									1	5.9									1	0.585
191	Sleepy	2	2	1	1	1	4.3			1	1					2	7.3	2	7.3	9	5.263
192	Sorry					1	1			1	1									2	1.170
193	Stressed					1	4.3	1	1	2	2	4	8.9	1	1	4	**20**	1	6.3	14	8.187
194	Strong															1	6.3			1	0.585
195	Stubborn			1	1															1	0.585
196	Sulky											1	1							1	0.585
197	Super	1	5.9					1	1											2	1.170
198	Surprised	1	1	3	**12**	1	4.3	1	5.6	1	1	4	4	2	6.9	2	2	1	1	16	9.357
199	Suspicious									2	6.9									2	1.170
200	Sweet															1	6.3			1	0.585
201	Sympathetic																	1	1	1	0.585
202	Sympathy															1	1			1	0.585
203	Tense									1	1									1	0.585
204	Terrified	2	6.9	1	1		1	1	5.6	1	1	2	2			2	2	1	6.3	11	6.433
205	Thankful						1													1	0.585
206	Thinking	1	1																	1	0.585
207	Thoughtful					1	4.3					2	2			1	6.3			4	2.339
208	Threatened											2	4.4							2	1.170
209	Tired	2	6.9			6	**13**			2	6.9	3	5.4	2	12	3	14	1	1	19	11.111
210	Tiredness									2	6.9									2	1.170
211	Tough					1	1													1	0.585
212	Truthful			1	1															1	0.585
213	Uncomfortable									1	1					2	7.3			3	1.754
214	Understood									1	1									1	0.585
215	Underwhelmed															1	6.3			1	0.585
216	Ungrateful															1	1			1	0.585
217	Unhappy			2	6.6	1	1	2	6.6	7	**17**	4	11	2	6.9	2	2	2	13	22	12.865
218	Unwell															1	1			1	0.585
219	Unwilling											1	1							1	0.585
220	Upset	2	6.9			4	**11**	3	12	2	2	4	6.4	7	22	5	16	8	29	35	20.468
221	Useful															1	1			1	0.585
222	Vain									1	5.9									1	0.585
223	Weak					1	1													1	0.585
224	Worked up															1	6.3			1	0.585
225	Worried	3	**13**	4	8.6	3	3	5	14	5	9.9	2	4.4	4	19			6	11	32	18.713
Total number of words		112		126		154		153		166		283		178		223		190			
Female mean		7.3		8.5		7.4		7.5		9.9		11.1		10.9		12.6		12.3			
Male mean		6.0		5.8		5.8		9.8		9.6		10.0		10.1		12.2		11.5			

Emboldened items represent earliest age at which word occurs in ≥ 10% of responses.

Items that occurred in at least 10% of the child responses are highlighted in bold within the table only in the youngest age group where this occurs. For example, ‘angry’ occurs in 51% of the children’s responses (at age 5–5.11) and continues to meet the threshold of ≥ 10% across the age groups. By doing this, 43 words were identified which occurred in ≥ 10% of children’s responses in at least one age group. *N* = 9 words (angry, embarrassed, excited, happy, hungry, petrified, sad, scared, worried) emerged for the first time in age group 5–5.11 years; *N* = 2 words (confused, surprised) emerged in ≥ 10% of child responses at aged 6–6.11; *N* = 4 words (lonely, shocked, tired, upset) emerged at 7–7.11; *N* = 3 words (annoyed, disappointed, okay) at 8–8.11; *N* = 6 words (bored, curious, grumpy, joyful, nervous, unhappy) at 9–9.11; *N* = 3 words (anger, depressed, fear) at 10–10.11; *N* = 5 words (cautious, hate, jealous, love, sick) at 11–11.11; *N* = 8 words (aggravated, amazed, cheerful, depression, disgusted, joyous, overwhelmed, stressed) at 12–12.11; *N* = 3 words (anxious, calm, distraught) emerging at 13–13.11.

Of the items that achieved ≥ 10% threshold, the majority either started as a common response in a younger age group and remained a common response across older age groups; e.g., (angry, embarrassed, excited, happy, sad, scared, worried) or gradually increased in prevalence from fewer responses in younger age groups and becoming more common in older age groups, for example, ‘anxious’ which started with an occurrence of 0% (5–5.11) and ended at 17% (13–13.11). Only two items started with a higher prevalence in a young age group, before diminishing, this was the word ‘hungry’ 13% (5–5.11) which diminished to between 0 and 5.6% over the remaining age groups, and ‘petrified’ starting at 12% (5–5.11) and diminishing to between 0 and 5.6% in the older age groups.

### Exploration of factors relevant to the future development of this novel measure

The data for both EVER Measure tasks (REV and WA:Emo) were scrutinized for factors which could prove important when developing this tool for future use.

Firstly, duplicated target words in the REV task were compared. The data indicated that ‘worried_1’ (not close visual stimuli) was correctly identified by ≥ 70% of children at age 5–5.11, while ‘worried_2’ (close stimuli) was identified correctly by ≥ 70% of children at 7–7.11. Therefore, the influence of the distractor affected the results in the direction which was expected, i.e., the identification of the target word was harder when the distractor was less easily discriminated. ‘Threatened-2’ (with no confounding distractor stimuli) was correctly identified by ≥ 70% at age 12–12.11, while ‘threatened_1’ (with morphologically confounding distractor stimuli) was identified correctly at 8–8.11. The influence of the distractor affected the results in the opposite direction from which we expected. Therefore, the identification of the target word ‘threatened’ was not made harder by the inclusion of stimuli with morphological confounding features.

Secondly, 71 word items were identified which occurred above the relevant threshold on either the WA:Emo (≥ 10%) or the REV (≥ 70%) data set *or both*. These were tabulated and the age at which the relevant percentage threshold was met was noted. Alongside the data for these measures, age of emergence as previously reported in parent and self-report scores (Baron-Cohen et al., 2010), were also included for comparison (see appendix 4). By so doing, differences between the three measures could be considered. Only five items were consistently identified as emerging within the same age bracket across the three measures of language of emotion (WA:Emo, REV and parent and self-report: Baron-Cohen et al., 2010): angry, excited, happy, worried (all 5 years) and surprised (6 years). However, 19 items reached threshold at a consistent age on two of the measures; afraid, sad, scared (5 years), confused (6 years), calm and shocked (7 years), disappointed (8 years), adoring and curious (9 years), fear and thoughtful (10 years), baffled and cautious (11 years) and overwhelmed (12 years). Several word items had a discrepancy of 3 years or more between measures: amazed, calm, cheerful, disgusted, embarrassed, hate, humiliated, love, mystified, startled. In addition, ‘sad’, considered to emerge at 5 years or before according the WA:Emo data and Baron-Cohen et al. (2010) was only identified correctly in the REV task by ≥ 70% of children at 8–8.11 years’.

## Discussion

Our findings indicate that The EVER Measure has potential for directly assessing the development of language of emotion across childhood (5–13 years). Currently, the tasks (and marking criteria) could be used to offer raw scores representing both the receptive and expressive domains. This has implications for research and ultimately clinical assessment. The large dataset also contributes to broader understanding of how emotion vocabulary emerges across childhood. Importantly, it offers preliminary new data on the emergence of emotion labels in middle-childhood, where previously no such direct assessment data had been available. Limitations to the findings and the measure itself are discussed below and will guide future research as well as iterations of its development.

### Proof of concept for The EVER Measure

Both subtests within The EVER Measure (REV and WA:Emo) demonstrated emotion vocabulary scores which increased in line with child age. By so doing, these findings indicate the potential of The EVER Measure as a tool for representing emotion vocabulary development between 5 and 13.11 years. Age was the only significant predictor of scores, with neither sex nor testing procedures affecting outcomes. Sex had been expected to be a significant predictor, based on accounts of typically-developing females out-performing males on certain tests of this type (Goddard et al., 2014; Kauschke et al., 2016; Sturrock et al., 2019). However, in terms of the *number* of accurate word items recognized or generated, this study found no such sex difference. There is thought to be a group advantage for females in terms of emotional awareness, particularly when this is assessed using measures reliant on linguistic content (Wright et al., 2018) and this might be due to differences in the amount of emotional conversation directed at girls by parents (Fivush et al., 2000). It is possible that the amount of emotion words a child knows or uses does not differ between sexes. It is possible that sex differences may be demonstrated through a different type of analysis, for example, an item-by-item analysis of word emergence comparing females’ or males’ vocabulary at different ages. The EVER Measure could provide a psycholinguistic assessment with the capacity to explore this relationship in larger samples.

Through a detailed exploration of data from the REV measure it was possible to determine that target words were representative of ability over the full range of ages assessed, with no ceiling effects at 13.11 years. Targets chosen appeared to emerge in a largely chronological fashion as predicted by findings from Baron-Cohen et al. (2010), although exact alignment did not occur and is discussed below. The WA:Emo task showed a broad distribution of newly emerging words (above threshold) across *all* age groups. One hundred and seventy eight word items on the WA:Emo did not reach threshold in this population, suggesting that this task has potential for representing development for children 13.11 years and above. On the REV measure only three word items (humiliated, frustrated, embarrassed) failed to reach threshold of ≥ 70% for emergence. As socio-emotional development is thought to continue into the early twenties (Blakemore and Mills, 2014; Bazhydai et al., 2019; Grosse et al., 2021) it would be important to track emotional labeling along the same period. It is likely that additional items would need to be included in the REV to avoid reaching ceiling in older childhood/adolescent groups.

The data also show moderate positive correlation between The EVER Measure subtests and matched measures of general vocabulary in receptive (BPVS) and expressive (WA) domains. It would be expected that emotional labeling and general vocabulary ability would develop in synthesis (Lindquist et al., 2015) and so tools measuring these phenomenon would reflect that relationship with positive correlation of findings. In this way the current study supports the development of The EVER Measure as a tool for identifying language of emotion development across age groups. Due to the co-linearity between general language and emotion vocabulary scores, this variable was not included as a possible predictor of language scores in our linear regression. However, it would be interesting to explore the relative influence of basic language versus emotional awareness on EVER scores in future research and additionally consider whether certain clinical groups demonstrate a disparity in this relationship. This would offer further insights into the relationship between language, emotional labels and emotional awareness (Hobson et al., 2019). Currently the direction of influence between emotion labeling and emotional awareness is poorly understood (Forrest et al., 2021). A new means of measuring this would contribute to exploring features in conditions such as alexithymia or in clinical groups, e.g., with emotional dysregulation.

### Understanding of emotion vocabulary acquisition across childhood

This study has a relatively large sample size (*n* = 171), yielding 6,669 data points for 39 receptive word items and a further 1,585 data points for 225 expressive word items. Importantly it obtains emotion vocabulary data across a previously under-researched population in middle childhood (5–13.11 years). These findings, therefore, provide a sizable contribution to the understanding of emotion language development in middle childhood. While there are some limitations to the use of novel measures in collecting data (discussed below), our findings could be used to form the basis of hypothesis making in related research. Middle childhood and pre-adolescence is potentially a critical period of emotional language growth. It is a time when social networks expand outside of core family and friends into complex new relationships within education and romantic arenas, essential for developing autonomy in adolescence (Wrzus et al., 2013). Currently, the field of emotion vocabulary research has focused on early emergence of words. Data from older children has been generated in a relatively small number of studies (Baron-Cohen et al., 2010; Beck et al., 2012; Bazhydai et al., 2019; Nook et al., 2020). The current study can expand on existing knowledge by offering data derived from direct assessments of both receptive and expressive tasks for children specifically in middle childhood.

The REV offered preliminary data on the emergence of receptive emotion vocabulary for a specific but representative subset of pre-selected items. Counts suggested that certain word items were established at age 5–5.11 years, e.g., happy, afraid etc. largely in line with Ekman’s 6 basic emotions (Ekman, 1992). These not only emerged early but also remained consistently well represented across all age groups. They also accounted for the most commonly correctly identified words within the whole data set. These words are commonly assessed in child populations (Bazhydai et al., 2019; Grosse et al., 2021) and their prevalence is to be expected. Other words, are not only later emerging but typically remain inconsistently understood even after they reached threshold. For example, ‘enthusiastic’ reached threshold at 9–9.11 years (15 children, 88.2% of this age group, correctly identifying this item). However, it continued to be recognized below saturation (highest occurrence was 93.8% at 13–13.11 years) across the age groups. In combination, the data seem to suggest that while some emotion words are both early emerging and commonly understood, others are both later emerging and inconsistently recognized, even in older cohorts of children. If vocabulary learning is experiential (Ponari et al., 2018), then it would be anticipated that understanding of certain words would vary according to this. Influencing factors might then include parental emotional literacy (Bozkurt Yükçü and Demircioğlu, 2021) and exposure to conversations about emotions more generally (Dunn et al., 1991). It is likely that many words will not reach 100% saturation across a child group and this is not something that should be looked for as a marker of year group competence.

By contrast (to the REV) the WA:Emo task was able to show word emergence from an unlimited potential pool. In the final count, 225 different correct items were identified by children, contributing a substantial data set to existing work in this area (Beck et al., 2012). In addition, the study methods resulted in a comprehensive and robust scoring matrix and emotion vocabulary database, making scoring easily replicable between studies and potentially resulting in data aggregation to create larger datasets.

The word-by-word analysis of the WA:Emo task, showed that, as a general trend, number of children generating each word increased incrementally across age groups. However, this was not always the case between adjacent year groups. This might be expected due to percentage calculations being based on relatively small actual numbers of word items. In only two cases did the word item begin with a high percentage then decrease to trace representation in subsequent age groups: ‘hungry’ (13% in 5–5.11 year old) and ‘petrified’ (12% at 5–5.11 years). ‘Hungry’ was included as a correct item in the database in line with evolutionary psychological theories of emotion (Izard, 2009). However, there is debate about its categorization as an emotion (Mulligan and Scherer, 2012) others suggest that it (along with ‘pain’, ‘thirst’) could be considered a primordial example of an emotion (Denton et al., 2009), indicative of its rather simplistic or base relationship to human experience compared to emotions such as fear and love. If hunger is thought to be a relatively base representation of the category emotion, this might explain why the current data showed that children used it less frequently across older age groups, where more sophisticated emotions were adopted. The high occurrence of the word ‘petrified’ was harder to interpret. Without comparative data to draw on, this word item seems rather mature for 5–5.11 year olds (both male). One interpretation might be that there is a language environment (a television show, book or game) accessed more commonly by this group which over represents this word item, enhanced its exposure and accelerating its acquisition (Gathercole, 2006). As stated, the percentage calculations were also prone to large fluctuations, which might over represent the significance of relatively minor changes in total numbers. Patterns of word emergence could be more accurately identified in larger sample sizes and the relevance and constancy of the ≥ 10% threshold could be better tested in a larger dataset of emotional vocabulary.

Analysis of WA:Emo data also provided a number of emotion word items which appeared to be emerging but not emerged by age 13.11. This will be useful new data to reflect on when selecting target items for the REV, and in particular, for capturing development in older children.

### Evidence for future development of The EVER Measure

The word item analysis for both the REV and the WA:Emo demonstrated some patterns worthy of note, with potential relevance for future iterations of The EVER Measure. These are briefly discussed below.

A comparison between the REV and WA:Emo scores was supplemented by comparison with the data from the Baron-Cohen et al. (2010) emotion questionnaire. While there was relatively little exact agreement between measures, many items on the REV occurred within one age bracket of the emotion questionnaire data, indicating some consistency between results. Differences might be attributed to the different testing procedures, for example, REV is a direct assessment tool and so taps in to the child’s actual ability rather than relying on reported measures (self and parent). As well as the usual reporter bias, receptive language is recognized as a difficult area to assess observationally (Conti-Ramsden and Durkin, 2012). This might indicate that ‘mystified’, for example, thought to emerge at 15–16 years (Baron-Cohen et al., 2010) might emerge younger with this study demonstrating emergence at 10 years old. A contributing factor could also include the slightly lower threshold for establishing emergence of ≥ 70% rather than 75% applied to REV. However, in the case of ‘mystified’, changing our threshold would not have brought the age of emergence into alignment and cursory explorations of applying this rule more generally did not improve agreement between the data. However, it would be important to establish some evidence-based principals for establishing this threshold in future iterations of the measure.

There were some examples of REV items emerging later than indicated on the emotion questionnaire. Large discrepancies (greater than one age bracket) in this direction included, choosing, embarrassed, frustrated, humiliated, interested and sad. In this case one of two factors could be influencing results, either mis-reporting of receptive abilities by parents, e.g., reporting a child knows a word when they do not, or confounds in the novel REV measure meaning children are not accurately identifying words that should be in their repertoire. This certainly seems to be the case with ‘sad’, which is commonly considered one of the basic emotions (Ekman, 1992; Matsumoto, 2001) and thought to emerge at under 5 years (Baron-Cohen et al., 2010). This was substantiated by 52% of children spontaneously generating this word in the WA:Emo measure.

One confound affecting the emergence of ‘sad’ in the REV data might be the closeness of the picture stimuli between the target word and its close distractor. Trial items in the measure, ‘worried_1’ and ‘worried_2’, demonstrated this confounding effect, with fewer children accurately identifying the target word when there was a close picture stimuli for the close distractor, with an age variation in meeting threshold of 5–5.11 compared to 7–7.11. While picture stimuli was piloted before testing and ‘sad’ stimuli was not considered overly close by adult respondents, it is possible that discrimination between the images may be more challenging for children. This indicates the need for trialing picture images on children (rather than adults) before inclusion in a next iteration of the task. It is possible that this contributes to the relatively late emergence of word items: choosing, embarrassed, frustrated, humiliated, interested, sad. Further evidence for this affect influencing age of emergence of at least the word item ‘sad’ in the REV measure comes from reviewing the combined scores of the correct (41.2%) and close distractor (58.8%), i.e., no children chose the distant distractor, in contrast to the majority of stimuli responses. The value of including a close and distant distractor is in offering rich data in the types of errors children make and whether they can make ball-park judgments about the word meaning. However, future iterations of this measure do require a systematic evaluation and rating of the closeness and distance of the distractor items by children to avoid confounding factors influencing results.

The stimulus item ‘threatened’ (which considered the effect of closeness of morphological features between stimuli; threatened versus threatening) showed no confounding effects, perhaps indicating that morphological factors are not significant when testing typically-developing children. Avoiding confounding factors of morphology in iterations aimed at testing language disordered children may be more important (Deevy and Leonard, 2018).

For most emotion words, WA:Emo data emerged later than either the REV or the emotion questionnaire data (Baron-Cohen et al., 2010) as would be expected for an expressive task (Hendriks and Koster, 2010), especially one where word recall incurs demands of not only word knowledge but also processing ability and verbal memory (Hedden et al., 2005). For two words, sad (as discussed above) and embarrassed, the word emerged in the WA:Emo task before the REV which contrasts with the expected order of development. Data from WA:Emo found ‘embarrassed’ to emerge at 5–5.11 year olds compared to not reaching threshold in the REV measures. Parent reports using the emotion questionnaire place emergence at 7–8 years, supporting neither of this study’s findings (Baron-Cohen et al., 2010). In this case the word ‘embarrassed’ might be singularly poorly represented in the current dataset. It is worth noting that ‘embarrassed’ (alongside ‘happy’) is one of the example words used in the introduction to the WA:Emo measure, potentially priming children sufficiently to increase the age of emergence thereby impact child scores. To avoid this type of influence in the WA:Emo measure, future iterations of the tool should use example words in the task introduction that emerge before 5 years, e.g., happy and angry. The item in the REV measure may be prone to under-recognition by the children perhaps due to relatively close distractor stimuli. This could be addressed in further development of the measure.

Possible effects of differences in BPVS scores across age groups were considered. In the WA:Emo data set there were fewer items emerging for the first time in the age bracket 6–6.11 (*n* = 2) compared to 7–7.11 (*n* = 4) as might be expected by the skew in BPVS scores. However, it was also anticipated that there would be greater numbers of newly emerging items in year group 11–11.11 compared to 12–12.11. However this was not the case; *n* = 5 compared to *n* = 8. The REV demonstrated only subtle differences between the number of words that emerged at 6–6.11 years old (*n* = 3) and 7–7.11 years (*n* = 4) but elevated scores at age 11–11.11 (*n* = 6) compared to 12–12.11 (*n* = 3). In combination, the data here is inconclusive and it is not possible to say whether BPVS scores across age groups was an influence in determining the age of emergence of words. It was not possible to control standard scores on this measure because of the use of two different version of the BPVS disproportionately in different year groups. This is a limitation in the study. However, inconclusive findings in year group analysis may support the notion that the elevated BPVS scores in 11–11.11 year olds may be more due to BPVS variation in calculating standard scores than actual differences in year group ability.

Finally, the WA:Emo task resulted in the creation of a comprehensive scoring matrix and emotion database. Currently, there is no standardized measure of word generation for either non-emotional or emotional categories. This is possibly due to the wide array of words likely to be generated and therefore difficulties in establishing consistency between scorers in designating items correct or incorrect. The matrix in this study was established through an iterative process, with multiple scorers contributing to refinement of the protocol and database through discussion of key items, with reference to the literature and consensus reaching. The final matrix produced strong agreement between novel markers in its final iteration. This process was repeated for the emotion protocol as well as the non-emotion categories (animals, food and occupations). In addition, an extensive database of emotion wards was generated, collapsing items from a review of the literature (Storm and Storm, 1987; Baron-Cohen et al., 2010; Cowen and Keltner, 2017) with team-based discussion on an item-by-item basis. Commonly occurring errors for the emotion category, such as ‘crazy’ or ‘funny’, were also cataloged. With further development this will offer a useful accompaniment to The EVER Measure, improving speed and consistency of marking on the WA:Emo task and potentially making WA:Emo a valuable tool for clinical and research application.

### Limitations

One of the aims for this study was to identify limitations within the existing EVER Measure, and by doing so, inform its future development. To that end, limitations of the measure itself have been considered extensively throughout this paper. Key points include the need for a systematic re-evaluation of picture stimuli, including closeness of distractors by children rather than adults, to determine appropriacy of all images and reduce confounding factors in the REV measure. Similarly, use of examples in the WA:Emo should not include items thought to emerge within the ages being tested. However, despite the need for some refinement in our stimuli these tools provided data on the emergence of emotion vocabulary across the age groups as defined, and do contribute to our understanding of emotional labeling in a middle childhood population. A future study would aim to check validity and reliability using tests of internal consistency in the REV.

Covid regulations limited recruitment and altered testing procedures already embarked upon in earlier data collection. However, detailed analysis of word emergence on a year-by-year basis did not provide conclusive evidence of a skew in the data and linear regression showed no predictive effect of testing procedure on EVER scores. A future study would curtail variation in the use of measures and procedures and would be delivered in a face-to-face setting, allowing for a lengthier battery of measures that could explicitly rule out language, social communication and intellectual disorders and ensure greater consistency between age group demographics.

This was a large population study providing appropriate sample sizes for correlation analysis and linear regression, our two main forms of analysis for evaluating EVER. However, when considering a word-by-word analysis at year group level, the data was reduced to smaller numbers, particularly in the WA:Emo task where children generated responses spontaneously from a large potential pool of items. A larger sample size would allow us to undertake a category analysis of word emergence between age groups and evaluate the use of the ≥ 10% threshold for the WA:Emo tool.

### Conclusion and future research

The EVER Measure demonstrated proof of concept for exploring the development of emotion vocabulary across the ages 5–13.11 years. This is a population where little is understood in terms of the development of emotional labels. However, it is a critical group for further exploration, with emotional and social demands rising steeply during this time. Emotional labeling is linked to improved emotional regulation (Lieberman et al., 2011; Kircanski et al., 2012) and having a direct assessment would be an important tool for evidencing this relationship within typically developing and clinical groups.

The REV and WA:Emo showed consistency with matched basic language measures and age was found to be the most significant predictor of scores. Additionally, word-by-word analysis was able to provide new preliminary data on the age of emergence for certain words. Although further refinement of the tool is indicated, the current findings suggest that both tasks can provide rich data pertinent to research in emotional vocabulary emergence over time. In this way it will contribute to the wider knowledge in this area and can inform hypothesis testing on ability in middle childhood.

The middle and longer term aims are to develop this tool in line with findings and establish validity and normative data on both subtests. Ultimately, The EVER Measure could have clinical application in assessing areas of strength and weakness in older children with neurodevelopmental and mental health conditions. The WA:Emo scoring matrix and emotion database is an integral part of that package, offering a structure for achieving consistent scoring. Establishing suitability and consistency of marking with clinical practitioners would be an important stage in future research.

## Data availability statement

The original contributions presented in the study are included in the article/Supplementary material, further inquiries can be directed to the corresponding author.

## Ethics statement

The studies involving human participants were reviewed and approved by The South West – Central Bristol NHS Research Ethics Committee, The University of Manchester Ethics Committee and The University of Bath Department of Psychology Ethics Committee. Written informed consent to participate in this study was provided by the participants’ legal guardian/next of kin.

## Author contributions

AS and JF devised the research questions and methodology, undertook and oversaw the research data collection, analyzed the data, and contributed equal parts to editing. AS lead on the writing of the current manuscript. The test measures were developed as part of AS’s PhD for which JF and CA were supervisors. Both authors contributed to the article and approved the submitted version.

## Conflict of interest

The authors declare that the research was conducted in the absence of any commercial or financial relationships that could be construed as a potential conflict of interest.

## Publisher’s note

All claims expressed in this article are solely those of the authors and do not necessarily represent those of their affiliated organizations, or those of the publisher, the editors and the reviewers. Any product that may be evaluated in this article, or claim that may be made by its manufacturer, is not guaranteed or endorsed by the publisher.

## Footnotes

^1^ https://www.autismresearchcentre.com/
