# Supplementary material for: Preliminary data on the development of emotion vocabulary in typically developing children (5–13 years) using an experimental psycholinguistic measure
Source: Front Psychol. 2023 Jan 31;13:982676. doi: 10.3389/fpsyg.2022.982676 (PMC9928212; doi:10.3389/fpsyg.2022.982676)
Supplement: Supplementary file 1 [file Data_Sheet_1.docx]

**Appendix 1: Table of target words and close distractors for the REV**

| Appendix 1: The 39 target emotion words included in the Receptive Emotion Vocabulary Task, close distractor and their position over the 4 box image layout (A-D, left to right top to bottom) | | | | |
| --- | --- | --- | --- | --- |
| Item Number | Target word | Position | Close distractor | Position |
| Practice | Grumpy | B | - | - |
| 1 | Happy | A | Friendly | D |
| 2 | Sad | B | Worried | D |
| 3 | Afraid | C | Disgusted | B |
| 4 | Excited | D | Happy | C |
| 5 | Worried_1 | C | Sad | B |
| 6 | Interested | B | Frustrated | D |
| 7 | Angry | B | Worried | A |
| 8 | Surprised | D | Interested | B |
| 9 | Disgusted | C | Angry | B |
| 10 | Confused | B | Worried | D |
| 11 | Calm | A | Friendly | B |
| 12 | Threatened_1 | A | Surprised | B |
| 13 | Proud | D | Overjoyed | C |
| 14 | Embarrassed | C | Afraid | B |
| 15 | Thoughtful | D | Confused | C |
| 16 | Overjoyed | C | Surprised | D |
| 17 | Guilty | C | Unfriendly | A |
| 18 | Worried_2 | C | Overwrought | B |
| 19 | Amused | D | Friendly | A |
| 20 | Startled | B | Anxious | C |
| 21 | Frustrated | B | Unfriendly | C |
| 22 | Affectionate | D | Friendly | A |
| 23 | Gloomy | B | Worried | A |
| 24 | Enthusiastic | A | Happy | D |
| 25 | Stubborn | B | Baffled | C |
| 26 | Threatened_2 | A | Surprised | B |
| 27 | Distant | C | Embarrassed | A |
| 28 | Determined | A | Aggressive | C |
| 29 | Devastated | D | Irritated | A |
| 30 | Adoring | A | Kind | D |
| 31 | Baffled | A | Unsure | D |
| 32 | Humiliated | D | Anxious | B |
| 33 | Contemptuous | D | Unfriendly | A |
| 34 | Choosing | C | Guilty | A |
| 35 | Intimidated | B | Worried | C |
| 36 | Mystified | A | Confused | C |
| 37 | Empathic | C | Enthusiastic | D |
| 38 | Indifferent | D | Stubborn | A |
| 39 | Stern | A | Confused | C |
