# Supplementary material for: Preliminary data on the development of emotion vocabulary in typically developing children (5–13 years) using an experimental psycholinguistic measure
Source: Front Psychol. 2023 Jan 31;13:982676. doi: 10.3389/fpsyg.2022.982676 (PMC9928212; doi:10.3389/fpsyg.2022.982676)
Supplement: Supplementary file 2 [file Data_Sheet_2.docx]

**Appendix 2: Scoring matrix for the WA (animals, food, occupations)**

**Guidance for Identifying Correct Items**

*Order for checking correctness*

1. Check against Oxford English Dictionary (OED) definition
2. Check on Google to determine if they are new or emerging words or word phrases
3. Check against authors’ list of rules (see below)

*A correct item entails:*

- Word or cluster of words that are in the dictionary alongside the correct meaning
- Word or cluster of words that are found in a Google search – with information identifying them as having the meaning intended under the category heading
- Correct items can include very low frequency items
- Correct items cannot be repetitions
- Super-ordinates and sub-ordinates are counted as correct
- The item should be phonetically accurate (not a close approximation, e.g., robsvokowski hamster)
- The item should be morphologically accurate (e.g., Olympic gymnastic, inamused, would not be accepted) – some exceptions may be allowed for common grammatical immaturities, for example, over generalisation of regular pluralisation rules in irregular nouns, e.g., gingerbread mans

**Specific information per category:**

**Animal Category**

Animal definition:

A living organism which feeds on organic matter, typically having specialized sense organs and a nervous system and able to respond rapidly to stimuli; any living creature, including man. (OED)

Part of the animal kingdom, a group of multicellular eukaryotic organisms (Britannica)

- Can be broadly defined as a mammal, bird, reptile, insect, fish
- Cannot be single cell organisms, germs, bacteria, plants, human-like, robots etc. (these are classed as intrusions)
- Can include large super super-ordinate categories, e.g., vertebrates

**Food category**

Food definition:

Any nutritious substance that people eat or drink in order to maintain life and growth (OED)

- Can include food brand names
- Can include drinks
- Can include super-super ordinates, e.g., carbohydrates
- Frequency of items may vary according to culture (e.g., dog), but should be marked correct
- When a type of meat is said, the word should refer to the food, not the animal (where applicable), e.g., beef, not cow. If the animal word is used, score 0, unless there is no other word used to refer to the meat (e.g., chicken).
- Can include condiments
- Different examples of the same basic food item, e.g., sandwich, can be counted, e.g., cheese sandwich, watermelon gum, as this is an example of attributive noun rather than an adjective, but also see error rule below

**Occupations category**

Occupation definition:

A person's usual or principal work or business, especially as a means of earning a living (dictionary.com)

A particular action or course of action in which a person is engaged, esp. habitually; a particular job or profession; a particular pursuit or activity (OED)

- Can include conclusions to the statement ‘I work at ...’, ‘I work in...’ or ‘I am a ...’ and may include a vehicle; e.g., ambulance
- Correct items can include a superordinate category, e.g., I work in the NHS.
- Can include both male and female version, e.g. actor and actress
- Inherited titles are not correct: including monarchy

**Errors can include neologisms, intrusions, and repetitions**

- *Neologisms* – a made up word or novel cluster of words (not found in a dictionary or Google search)
  - Made up word: archerist
  - Novel cluster of words; ‘lactic reef shark’ possibly means arctic reef shark; ‘singing owl’ not real bird
- *Intrusion* – a real word or cluster of words (found in the dictionary or Google search) but not from the correct category
  - An implied link is not sufficient; e.g. ‘dinosaurs’ under food might refer to potato shapes which look like dinosaurs, but simply to say dinosaurs is insufficient
  - Animals errors include extinct and fictitious animals; dinosaurs, dodo
  - Summary items are intrusions; e.g., all the jobs in the world
- *Repetition* (uncorrected)
  - Includes repetition of nouns where only the qualifier is changed, e.g. apple/red apple, gum/watermelon flavour gum.
  - Where a noun phrase is made of a main noun and an attributive noun, this would be considered correct, even if part of the phrase has been repeated, e.g., child says ‘cheese’ then ‘cheese sandwich’ both are correct – score ‘2’. If the child has said both parts before, e.g., ‘cheese’, ‘sandwich’, ‘cheese sandwich’ then this is counted as ‘2’ for the novel elements and not ‘3’ for the individual tokens. In this case only novel items are scored, not multiple, reused items.
  - A repetition is a repetition when the noun remains the same, and the only thing that changes is the adjective
