# Supplementary material for: Preliminary data on the development of emotion vocabulary in typically developing children (5–13 years) using an experimental psycholinguistic measure
Source: Front Psychol. 2023 Jan 31;13:982676. doi: 10.3389/fpsyg.2022.982676 (PMC9928212; doi:10.3389/fpsyg.2022.982676)
Supplement: Supplementary file 3 [file Data_Sheet_3.docx]

**Appendix 3: Scoring matrix for the WA:Emo**

**Emotions definition**:

A feeling derived from one's circumstances, mood, or relationships with others. Instinctive or intuitive feeling as distinguished from reasoning or knowledge (OED)

A complex event that involves an evaluative component (e.g., appraising the situation), a physiological component (e.g., autonomic reaction), a phenomenological component (e.g., amygdala response), an expressive component (e.g., facial expression), a behavioural component (e.g., higher level response to emotion; running away, caressing, etc.) (Stanford Encyclopedia of Philosophy)

*Additionally*

- Emotions can vary in typicality; ‘fear’ is more typical than ‘awe’, but both are correct for the purposes of scoring this project
- Some emotions are commonly rated as ‘borderline’ by native speakers, e.g., that they border between emotions and cognitive mental states. Due to philosophical differences identified in the literature, borderline cases will be included as *correct* in this scoring.

*Item checking is s three part process:*

- 1. Items should be checked against the author’s catalogue of emotions: master sheet
  2. Items should be checked to see if they are included in the catalogue of emotions: synonym sheet (synonyms derived directly from the master sheet)
  3. If they are not included in either sheet, search the original word in the dictionary for its synonyms. If a synonym for the word is in the catalogue of emotions: master sheet, it can be counted as correct

**Newly agreed synonyms can be included in the synonym list with a note as to why they are included**

*Details of marking to consider*

- Some synonyms may be phrases, e.g., ‘over the moon’. These should be common phrases, e.g., occurring in the OED, and then checked against the catalogue of emotions, dictionary etc.
- Synonyms could be added to the catalogue of emotions after discussion with the team
- Adverbs may be counted if the root word is not a repetition of a pre-existing word, e.g., hopeful is fine, hopefully is fine, but only one is allowable in the same child’s list of words.
- Discount any other rules from previous scoring instruction documents. These may still be used in team discussions about entering words on to the catalogue of emotion database only

*Errors might include: Neologisms, intrusions, repetitions*

- *Neologisms* – a made up word or novel cluster of words (not found in a dictionary or Google search)
  - Made up word: unsad
  - Novel cluster of words; ‘happy in tears’
- *Intrusion* – a real word or cluster of words (found in the dictionary or Google search) but not from the correct category
  - An implied link is not sufficient; e.g. ‘playing with cats’
  - Summary items are intrusions; e.g., every emotion in the world
  - Emotion word errors should not be in the catalogue of emotions: master sheet, nor synonyms of words in the catalogue of emotions: master sheet.
- *Repetition* (uncorrected)
  - Includes repetition of nouns where only the qualifier is changed, e.g. wildly happy, stupidly happy
  - Where a noun phrase is made of a main noun and an attributive noun, this would be considered correct, even if part of the phrase has been repeated, e.g., child says ‘angry’ then ‘tired angry’ both are correct – score ‘2’. If the child has said both parts before, e.g., ‘angry’, ‘tired’, ‘tired angry’ then this is counted as ‘2’ for the novel elements and not ‘3’ for the individual tokens. In this case only novel items are scored, not multiple, reused items.
  - A repetition is a repetition when the noun remains the same, and the only thing that changes is the adjective, e.g., happy, very happy
