# Supplementary material for: Preliminary data on the development of emotion vocabulary in typically developing children (5–13 years) using an experimental psycholinguistic measure
Source: Front Psychol. 2023 Jan 31;13:982676. doi: 10.3389/fpsyg.2022.982676 (PMC9928212; doi:10.3389/fpsyg.2022.982676)
Supplement: Supplementary file 4 [file Data_Sheet_4.docx]

**Appendix 4: table of between measure comparisons of emotion vocabulary emergence**

| Appendix 4. Showing agreement between word age of emergence as indicated by the WA:Emo and REV direct assessment and findings from child and parent report measures (Baron-Cohen, et al., 2010) | | | | | | | |
| --- | --- | --- | --- | --- | --- | --- | --- |
|  | WA: Emo task | REV task | Child / parent report |  | WA:Emo task | REV task | Child / parent report |
| *Adoring* | - | 9 | 9-10 | *Guilty* | - | 11 | 9-10 |
| *Affectionate* | - | 10 | 11-12 | *Happy* | 5 | 5 | 4-6 |
| *Afraid* | - | 5 | 4-6 | Hate | **11** | - | **4-6** |
| Aggravated | 12 | - | - | *Humiliated* | - | **14+** | **9-10** |
| Amazed | **12** | - | **7-8** | Hungry | 5 | - | - |
| *Amused* | - | 11 | 9-10 | *Indifferent* | - | 13 | - |
| Anger | 10 | - | - | *Interested* | - | 9 | 4-6 |
| *Angry* | 5 | 5 | 4-6 | *Intimidated* | - | 10 | - |
| Annoyed | 8 | - | 4-6 | Jealous | 11 | - | 7-8 |
| Anxious | 13 | - | 9-10 | Joyful | 9 | - | - |
| *Baffled* | - | 11 | 11-12 | Joyous | 12 | - | - |
| Bored | 9 | - | 4-6 | Lonely | 7 | - | 4-6 |
| *Calm* | **13** | **7** | 7-8 | Love | **11** | - | **4-6** |
| Cautious | 11 | - | 11-12 | *Mystified* | - | **10** | **15-16** |
| Cheerful | **12** | - | **7-8** | Nervous | 9 | - | 7-8 |
| *Choosing* | - | 9 | 4-6 | Okay | 8 | - | - |
| *Confused* | 6 | 6 | 7-8 | *Overjoyed* | - | 7 | 9-10 |
| *Contemptuous* | - | 11 | 13-14 | Overwhelmed | 12 | - | 11-12 |
| Curious | 9 | - | 9-10 | Petrified | 5 | - | - |
| Depressed | 10 | - | 7-8 | *Proud* | - | 5 | 7-8 |
| Depression | 12 | - | - | *Sad* | **5** | **8** | 4-6 |
| *Determined* | - | 11 | 9-10 | Scared | 5 | - | 4-6 |
| Devastated | - | 9 | - | Shocked | 7 | - | 7-8 |
| Disappointed | 8 | - | 7-8 | Silly | 11 | - | - |
| Disgusted | **12** | **5** | **9-10** | *Startled* | - | **7** | **11-12** |
| Distant | - | 9 | 11-12 | *Stern* | - | 9 | 0 |
| Distraught | 13 | - | - | Stressed | 12 | - | - |
| Embarrassed | **5** | **14+** | **7-8** | *Stubborn* | - | 12 | - |
| Empathic | - | 12 | - | *Surprised* | 6 | 6 | 4-6 |
| Enthusiastic | - | 9 | 11-12 | *Thoughtful* | - | 10 | 9-10 |
| Excited | 5 | 5 | 4-6 | *Threatened* | - | 8 or 12 | 9-10 |
| Fear | 10 | - | 9-10 | Tired | 7 | - | - |
| Frustrated | - | 14+ | 11-12 | Unhappy | 9 | - | 4-6 |
| Gloomy | - | 6 | 9-10 | Upset | 7 | - | 4-6 |
| Grumpy | 9 | - | 4-6 | *Worried* | 5 | 5 or 7 | 4-6 |
| Italicised = items from the REV task; highlighted = agreement on age between measures; bold = more than three years between age of emergence as reported from the individual test items.  [-] denotes an items that was not tested or did not occur in the data set. | | | | | | | |
